# Variation in Human Recombination Rates and Its Genetic Determinants

**DOI:** 10.1371/journal.pone.0020321

**Published:** 2011-06-17

**Authors:** Adi Fledel-Alon, Ellen Miranda Leffler, Yongtao Guan, Matthew Stephens, Graham Coop, Molly Przeworski

**Affiliations:** 1 Department of Human Genetics, University of Chicago, Chicago, Illinois, United States of America; 2 Department of Statistics, University of Chicago, Chicago, Illinois, United States of America; 3 Department of Evolution and Ecology and Center for Population Biology, University of California Davis, Davis, California, United States of America; 4 Department of Ecology and Evolution, University of Chicago, Chicago, Illinois, United States of America; 5 Howard Hughes Medical Institute, University of Chicago, Chicago, Illinois, United States of America; Institut Pasteur, France

## Abstract

**Background:**

Despite the fundamental role of crossing-over in the pairing and segregation of chromosomes during human meiosis, the rates and placements of events vary markedly among individuals. Characterizing this variation and identifying its determinants are essential steps in our understanding of the human recombination process and its evolution.

**Study Design/Results:**

Using three large sets of European-American pedigrees, we examined variation in five recombination phenotypes that capture distinct aspects of crossing-over patterns. We found that the mean recombination rate in males and females and the historical hotspot usage are significantly heritable and are uncorrelated with one another. We then conducted a genome-wide association study in order to identify loci that influence them. We replicated associations of RNF212 with the mean rate in males and in females as well as the association of Inversion 17q21.31 with the female mean rate. We also replicated the association of PRDM9 with historical hotspot usage, finding that it explains most of the genetic variance in this phenotype. In addition, we identified a set of new candidate regions for further validation.

**Significance:**

These findings suggest that variation at broad and fine scales is largely separable and that, beyond three known loci, there is no evidence for common variation with large effects on recombination phenotypes.

## Introduction

In most sexually-reproducing species, including humans, recombination is crucial to the proper pairing and segregation of homologous chromosomes. Meiotic recombination events result from the formation and repair of double-strand breaks and appear to localize primarily to 1–2 kb «hotspots». At a subset of breaks, the repair leads to a crossover resolution, providing a physical connection between homologous chromosomes that aids in their correct segregation. In the absence of a backup mechanism, at least one crossover is required per chromosome to ensure proper disjunction. Too few crossovers can lead to aneuploidy and, more generally, errors in recombination can compromise genome integrity [1].

Given the functional importance of recombination, one might expect the process to be tightly regulated. In some respects it clearly is, as (in most organisms) numerous mechanisms act to ensure the occurrence of at least one crossover per chromosome and events are spaced farther apart and more evenly than expected by chance [2], [3]. But recombination is also surprisingly variable: differences among individuals are seen at every scale, from the single hotspot to the whole genome, with particularly pronounced variation in total genetic map length among human females [4], [5]. The regulation and rates of recombination can also evolve rapidly between species (e.g., [6], [7], [8]).

Even though recombination is subject to many layers of control, which likely buffer the effects of differences among individuals, some of this variation has phenotypic and fitness consequences. In humans, in particular, too little crossing-over or an abnormal placement of events is a leading cause of spontaneous miscarriage and of severe developmental disabilities [1]. Moreover, mothers with a higher mean crossing-over rate have slightly but significantly more children, indicating that recombination is under fertility selection in contemporary populations [9], [10]. More generally, there is a vast literature in evolutionary biology outlining the conditions under which changes in recombination can be indirectly favored because of its effects on the efficacy of selection (e.g., [11], [12], [13], [14]). To better understand the selective pressures acting on recombination, however, we need to know more about the nature of recombination rate variation and its determinants [15]. Among important questions: Which aspects of recombination rate variation are under genetic control? How many loci are involved? Do the same loci contribute to large and fine-scale variation? To what extent are the effects sex-specific? What pleiotropic roles do the genes play? Answering the questions will also advance our understanding of how recombination is regulated and highlight loci potentially underlying differences in fertility.

Over the past decade, pedigree studies have provided a first glimpse, revealing that in humans, as in other organisms, the mean recombination rate is influenced by genetic variation. Notably, the mean recombination rate in human females was estimated to have a broad-sense heritability of 0.3 based on sib-pairs [9]. This estimate is hard to interpret, however, as the imprecision of individual phenotypes will bias the estimate downwards, while the use of sib-pairs confounds maternal and genetic effects, potentially leading to an over-estimate of heritability (especially since recombination events are initiated when the future mother is a fetus, i.e., in utero). That there is some genetic contribution to variation in the mean recombination rate is clear, however, as this trait is associated with markers in RNF212 in an Icelandic population sample [16]. RNF212 is the homolog of ZHP-3, a gene required for crossing-over in *C. elegans* and which appears to play a role in restructing chromosome structure in response to crossing-over, thereby aiding in proper disjunction [17]. Interestingly, the RNF212 haplotype associated with increased recombination rates in males *decreases* rates in females. The mean recombination rate in females is also associated with Inversion 17q21.31, again in an Icelandic population [18]. Consistent with recent selection at or near these regions, SNPs at both RNF212 and Inversion 17q21.31 appear to show unusually high differentiation among populations [16], [18]. More recently, a study replicated these two loci in samples of primarily European ancestry and reported that four additional genes (KIAA1462, PDZK1, UGCG, NUB1) influence mean recombination rates in either males or females, in total accounting for ∼5% of the sex-specific population variance [19].

Studies have also characterized variation in recombination patterns at a finer-scale, in terms of the fraction of crossovers that occur in hotspots detectable in linkage disequilibrium (LD) data (henceforth «historical hotspot usage») [10], [20]. In a candidate gene study, historical hotspot usage was shown to be strongly associated with alleles in the zinc finger array of PRDM9 in a founder population of European descent [20], accounting for an estimated 18% of the population variance. Providing experimental support for the crucial role of PRDM9, a sperm typing study of 10 hotspots demonstrated that differences in the array lead to differential use of the hotspots [21]. PRDM9 was further shown to be associated with variation among Icelandic individuals in a related phenotype, the fraction of crossovers occurring in 10 kb regions that are highly recombinationally active compared to the genome average [22].

The zinc finger of PRDM9 is predicted to bind a 13-mer motif overrepresented in recombination hotspots relative to coldspots (henceforth «Myers motif») and allelic variants of the finger bind their predicted recognition motifs with the expected affinities in vitro [8], [20], [23], [24]. The Myers motif was estimated to play a role in ∼40% of human hotspots [23], [25], but may in fact influence most or all hotspots in the genome [20], [21]. Intriguingly, the zinc finger domain shows high levels of polymorphism within humans [21], [26] and has experienced rapid evolution both between humans and chimpanzees [8] and across a wide range of mammalian taxa [27], [28].

To learn more about variation in human recombination and its genetic determinants, we characterized variation in five aspects of the recombination process, estimated their heritabilities, and performed a genome-wide search for loci associated with differences among individuals at both broad and fine scales.

## Results

### Recombination phenotypes

To estimate recombination phenotypes, we used genome-wide genotyping data made available by the Framingham Heart Cohort Study (FHS) and the Autism Genetic Resource Exchange (AGRE) for a large number of pedigrees and focused on autosomes (454,934 and 390,671 SNP markers, respectively; see Methods). We considered all nuclear families of two or more children (since crossover locations cannot be inferred in trios without grandparental genotypes) and applied quality control filters to the data. In total, we were left with 1,154 male and 1,149 female parents (most of whom had two to three offspring) in which we could estimate recombination phenotypes. We inferred the location of crossovers in the offspring by the method of Coop et al. (2008) [10] and by a new Hidden Markov Model that we developed (see Materials S1 for details). Results were highly concordant between the two methods, so we proceeded with the results from the method of Coop et al. (2008). Given the dense markers, over 99% of crossovers were resolved to within 500 kb, and 17% and 24% of events were resolved to within 30 kb in AGRE and FHS, respectively.

We also considered genome-wide genotyping data from the Hutterites (HUTT), a founder population of European ancestry, of which we had previously analyzed a much smaller sample [10], [20]. These individuals are all embedded within a large pedigree, which is known. To infer crossover events, we broke up the data into 163 overlapping nuclear families, who have a median family size of 4 genotyped offspring. In these data, ∼19% of crossover events resolved to within 30 kb.

From the crossover calls, we estimated five phenotypes for each parent: **(i)** The mean rate of recombination, i.e., the genetic map length averaged across children. **(ii)** The telomere usage, i.e., the fraction of crossovers that occurred in the 20% most telomeric base pairs of each chromosome arm. **(iii)** The centromere usage, i.e., the fraction of crossovers that occurred in the 20% most centromeric base pairs of each chromosome arm. **(iv)** The «historical hotspot usage» (defined as in [10], [20]). Namely, we considered historical hotspot usage to be the genome-wide fraction of well-defined crossover events that overlap a historical recombination hotspot (i.e., one inferred from linkage disequilibrium data). For each individual, we estimated this fraction, α, using a maximum likelihood approach, considering well-defined crossovers to be those delimited to within 30 kb and using hotspots inferred from linkage disequilibrium data [24]. This approach corrects for the possibility of overlap by chance [10]. **(v)** «Myers motif hotspot usage». For a subset of hotspots that are identified from patterns of LD and well-localized, Myers et al. [23] estimated the probability that a given hotspot was caused by the 13-mer «Myers motif». As our recombination phenotype, we estimated the genome-wide fraction of crossover events in historical hotspots attributable to the Myers motif for each individual (see Materials S1).

**Figure 1:**
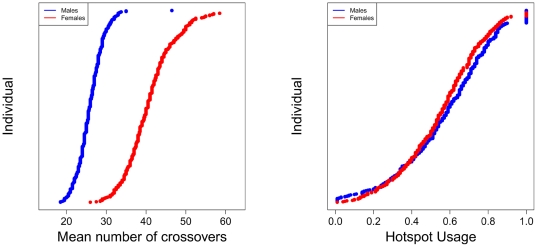
Variation in mean recombination rate and historical hotspot usage in the AGRE. Individuals are ordered within sex by their estimated phenotype, with males in blue and females in red.

**Figure 2:**
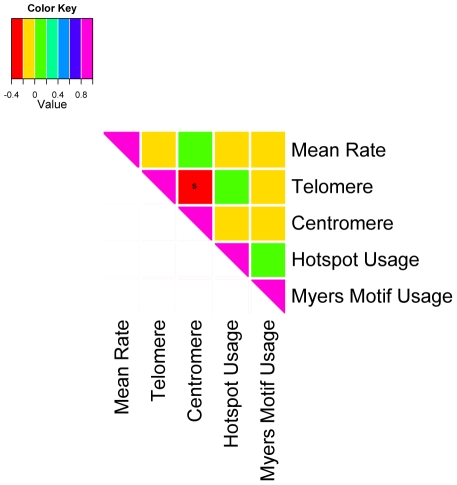
Correlation among the five recombination phenotypes in the AGRE. The strength of the correlation coefficient is color-coded; «s» indicates significance at the 5% level. Other than a negative correlation between telomere and centromere usage (p = 1.97×10^−9^), the five phenotypes are not significantly correlated with one another.

Variation in mean recombination rate and historical hotspot usage in the AGRE sample are shown in Figure 1 (for other phenotypes and samples, see Supplementary Figures 3–7 in Materials S1). Interestingly, historical hotspot usage and mean recombination rate are not significantly correlated (Figure 2), confirming the finding for a smaller set of Hutterites [10]. In fact, the only association among our five recombination phenotypes that is consistently significant across population samples is a negative relationship between telomere and centromere usage (see Supplementary Figure 8 in Materials S1 for FHS and HUTT results). Thus, the five phenotypes capture distinct aspects of recombination.

To estimate heritabilities of these five phenotypes, we took advantage of the HUTT pedigree structure (i.e., of the varying degrees of kinship), which allows us to obtain more precise estimates than would be possible with the equivalent number of nuclear families, and to obtain estimates of the additive genetic variance (i.e., the narrow sense heritability). We found that two of the five phenotypes were significantly heritable: the estimated narrow sense heritability was h^2^ = 0.23 (p = 0.011) for historical hotspot usage, almost identical to what we had estimated based on a smaller sample of HUTT [10] (see Supplementary Table 1 in Materials S1). In turn, h^2^ = 0.25 (p = 0.015) for female mean rate, an estimate consistent with the broad-sense heritability of 0.3 previously obtained from sib-pairs and potentially less confounded by maternal effects [9]. The heritability for the male mean rate, to our knowledge the first such estimate, was also marginally significant (h^2^ = 0.14; p = 0.078). We note that these heritabilities may be substantially under-estimated: while HUTT family sizes tend to be large, parents have a limited number of children, so there remains considerable imprecision in the measurement of recombination phenotypes, notably for historical hotspot usage.

**Table 1:** Strongest associations for the sex-specific mean recombination rate and for hotspot usage.

CHR	SNP	Gene	Left Gene	Right Gene	MAF (FHS)	MAF (AGRE)	P	FHS	AGRE
Male Mean Rate									
4*	rs11939380	RNF212	FGFRL1	SPON2	0.320	0.330	7.10×10^−16^	4.98×10^−13^	5.98×10^−4^
5	rs17542943	NA	MYO10	LOC285696	0.113	0.114	7.60×10^−7^	5.79×10^−5^	4.69×10^−3^
7+	rs11764733	NA	NUB1	WDR86	0.395	0.396	1.75×10^−6^	1.08×10^−4^	5.76×10^−3^
Female Mean Rate									
9	rs10985535	NA	TTLL11	NDUFA8	0.074	0.075	8.27×10^−7^	2.42×10^−5^	1.17×10^−2^
1	rs564636	OBSCN	C1orf69	TRIM11	0.325	0.298	1.39×10^−6^	1.47×10^−5^	2.98×10^−2^
10+	rs2505115	NA	KIAA1462	MTPAP	0.136	0.134	1.83×10^−6^	3.1×10^−4^	1.85×10^−2^
Historical Hotspot Usage (males and females, combined)									
5*	rs41502455	NA	PRDM9	CDH10	0.147	0.147	1.31×10^−8^	7.73×10^−7^	4.30×10^−3^
2	rs17011067	NA	TACR1	FAM176A	0.183	0.210	1.31×10^−6^	1.43×10^−4^	2.98×10^−3^
18	rs1864309	CCBE1	LMAN1	PMAIP1	0.451	0.418	1.60×10^−6^	4.83×10^−4^	9.57×10^−4^
Historical Hotspot Usage (males and females, combined, PRDM9 regressed)									
15	rs16972342	KIAA1199	FAM108C1	MIR549	0.062	0.052	4.95×10^−6^	1.02×10^−2^	3.99×10^−5^
18	rs1864309	CCBE1	LMAN1	PMAIP1	0.451	0.418	5.75×10^−6^	8.61×10^−4^	2.07×10^−3^
22	rs7284619	NA	ISX	HMGXB4	0.182	0.176	7.55×10^−6^	3.66×10^−4^	7.03×10^−3^

Provided are the chromosome, the rs number of the SNP with the lowest p-value in the region, the gene in which the SNP falls and the closest flanking genes. The minor allele frequencies in FHS and AGRE are given in columns «MAF (FHS)» and «MAF (AGRE)» respectively. The p-values are provided for the meta-analysis of FHS and AGRE in column «P», and for FHS and AGRE alone in columns «FHS» and «AGRE».

Loci identified by Chowdhury et al.

Other previously reported loci.

To investigate the extent to which the genetic basis of variation in recombination rates is sex-specific, we estimated the additive genetic variance components in a combined analysis of male and females of mean rates, standardizing the phenotype within each sex [29]. Both an autosomal and an X chromosome additive genetic variance component were included, but the estimated effect of the X chromosome was very close to zero. We found that the autosomal additive genetic variance of standardized mean genetic map length is 0.20 among females and 0.18 among males, very close to our estimates of narrow sense heritabilities (as expected, given that the rates were standardized within sexes). In contrast, the additive genetic covariance between male and female rates is only 0.03, suggesting that the additive genetic component of variation in genetic map length is largely sex-specific.

### Genome-wide association study

We sought to identify loci associated with variation in mean rate and historical hotspot usage using the FHS and AGRE population samples. To this end, we included X-linked as well as autosomal markers and imposed additional quality filters on the genotype data. To guard against spurious associations, we controlled for the number of genotyped children (which, by our estimation procedure, appeared to have a slight but significant artifactual effect on mean rates) and for cryptic population structure. We considered the two phenotypes as a quantitative trait in males, females and in the two sexes jointly (standardizing the phenotype within each sex). For each of these six tests, we performed a fixed-effects meta-analysis of the association test results from the FHS and AGRE samples for the intersection of 308,869 SNPs surveyed in the two studies [30]. We also imputed SNPs using all the population samples from the low coverage pilot of the 1000 Genomes Project [31] and the Hapmap CEU population as reference panels and tested for an association with 4.4×10^7^ SNPs [32], [33]. Finally, we tested for an association of SNPs with these two phenotypes in our much smaller HUTT sample, using a program that accounts for the relatedness of the sample [34] (see Materials S1 for details).

**Figure 3:**
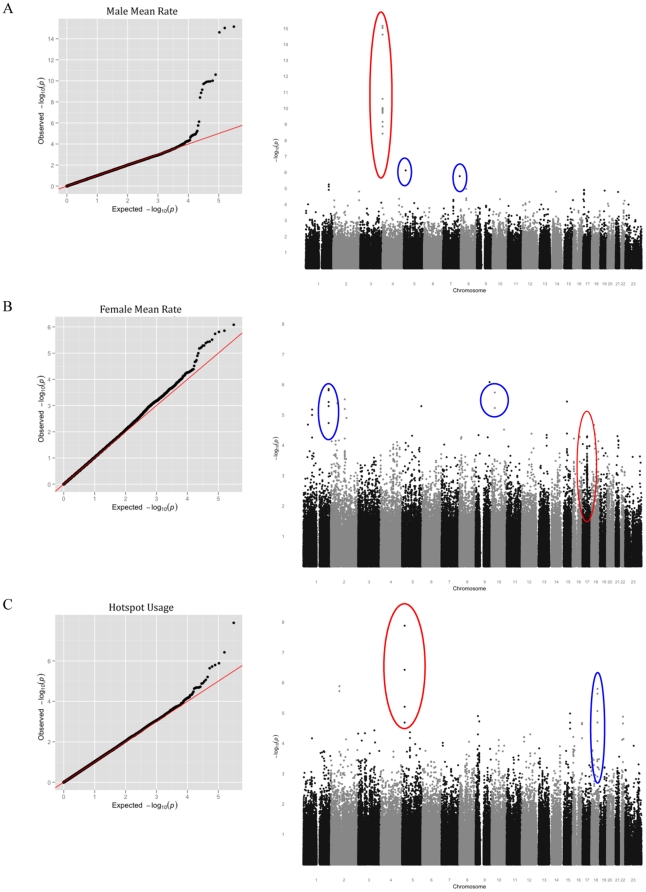
Results of the meta-analysis of FHS and AGRE for three recombination phenotypes. Each row consists of a Q-Q plot of observed against expected p-values (left panel) and a Manhattan plot showing the observed p-values across the genome (right panel). A. For the male mean rate. In the Manhattan plot, SNPs at RNF212 are circled in red and new candidate associations discussed in the main text are circled in blue. B. For the female mean rate. In the Manhattan plot, SNPs at or near Inversion 17q21.3 are circled in red and new candidate associations discussed in the main text are circled in blue. C. For the historical hotspot usage in the two sexes. SNPs near PRDM9 are circled in red and a new candidate association discussed in the text is circled in blue.

**Figure 4:**
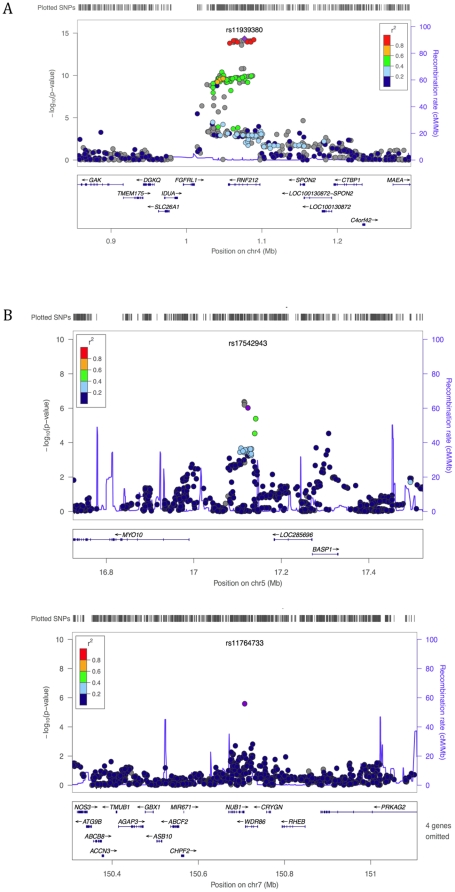
A close up of the association signal at previously reported and new candidate regions for male mean recombination rate. The figures show the p-values across the candidate regions for the genotyped and imputed SNPs plotted using the LocusZoom software [51] (only SNPs with an rs numbers are shown, but plots using all SNPs were qualitatively similar). The 1000 Genomes Project data [31] was used for the imputation in all LocusZoom figures, with the exception of Figure 4A for which HapMap data were used (as in this region LD patterns in the 1000 Genomes data were inconsistent with those from HapMap and the AGRE and FHS samples). The imputation-based approach uses a different test statistic than we employed in our analysis [32], so p-values can differ slightly from those reported in the main text. The focal SNP (with the lowest p-value) is plotted as a purple diamond; other data points are colored according to their r^2^ with the focal SNP; SNPs with missing linkage disequilibrium information are shown in grey. A. Association of male mean recombination rate with SNPs in RNF212. B. Top associations for male mean recombination rate.

Results of the meta-analysis of FHS and AGRE for sex-specific recombination rate are shown in Figure 3A–C. For male mean recombination rate, markers in RNF212 meet the cut-off for genome-wide significance (Figure 4A, p = 10^−15^ for the strongest association; see Table 1). Although our sample size is much smaller, RNF212 also has a low p-value in the HUTT (p = 4.14×10^−3^). Thus, the effect of this locus is confirmed in three population samples. In the FHS, it explains 7% of male variance in mean rates, with one allele estimated to add an average of 118 cM to the genetic map. We also replicated the association of RNF212 with female rates, to our knowledge for the first time (p = 2.15×10^−4^). Of note, the set of SNPs most strongly associated with male rates has no effect on female rates (the lowest p-value among them in females  = 0.162), whereas the set of SNPs associated with female rates show a weaker association in males (p = 1.379×10^−9^). This pattern suggests that, rather than a single causal SNP with antagonistic effects between sexes, there may be distinct causative SNPs in RNF212. Using the 1000 Genomes and Hapmap panels to impute untyped SNPs does not help to localize the causative allele(s), as the SNPs with the strong associations are seen throughout the gene (Figure 4A).

Although variation in RNF212 does not account for the heritability in male and female rates, no other loci meet the cut-off for genome-wide significance. Given the platform used for genotyping FHS and AGRE, we expect that the markers used in this study should be in high pairwise linkage disequilibrium (i.e., r^2^>0.8) with common SNPs in approximately two-thirds of the genome [35], [36]. Moreover, when we run power simulations taking into account the imprecision in phenotype measurement, we find that, so long as a common causative variation is present (or very well tagged) by SNPs on the array, we should have >95% power to detect effect sizes in males as large or larger as those reported for RNF212 at this significance level (Supplementary Figure 11 in Materials S1). (For the same absolute effect on the genetic map length, we have lower power to detect loci associated with variation in females than in males, because the length of the genetic map is more variable among females.) Thus, our findings indicate that, other than RNF212, there are no common loci with large effects on male mean recombination rates in approximately two-thirds of the genome.

Among the top associations with male mean rate, there are promising candidate regions, however. For example, the strongest new association signal is at SNP rs17542943 (p = 7.59×10^−7^; see Table 1), which lies in a primate conserved element that appears to be an enhancer based on ChIP-seq and H3K4me1 data (http://genome.ucsc.edu). Substantial LD (r^2^>0.2) extends out ∼50 kb (Figure 4B). The SNP lies 134 kb upstream of *Myosin-10*, a gene known to be involved in meiotic spindle formation in *Xenopus* [37] and to be expressed in human testes and in mouse spematocytes during Leptotene/Zygotene [38]–[39]. The next strongest association is 1 kb upstream of *NUB1* (p = 1.75×10^−6^, Figure 4B), as previously reported by [19]. This gene is expressed in human testes and during male meiosis in mice [19], [39].

**Figure 5:**
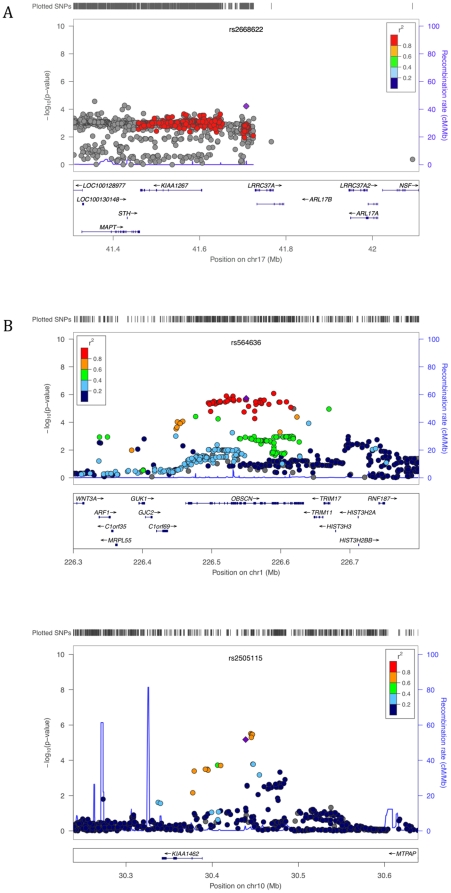
A close up of the association signal at previously reported and new candidate regions for female mean recombination rate. The figures were generated using the software LocusZoom [51], as described in the legend of Figure 4. A. Replication of association signal for female mean recombination rate near inversion 17q21.13. B. Top associations for female mean recombination rate.

In addition to RNF212, we replicated the effect of SNPs near Inversion 17q21.31 on female mean rate (lowest p-value  =  5.12×10^−5^, Figure 5A). The top SNP explains 1.45% of the female variance in this trait in FHS, with one allele estimated to add 124 cM on average to the genetic map. Among the strongest associations is SNP rs564636 (p = 1.39×10^−6^; see Table 1), which lies in an intron in the gene OBSCN, 113 kb from C1orf69 and 98 kb from TRIM11, in a region of high LD that extends as far as the gene HIST3H3 (Figure 5B). In the imputation analysis, several missense SNPs in OBSCN are in high LD and have low p-values. OBSCN, a member of a family of sarcomeric signaling proteins, is expressed in mouse male meiotic cells, suggesting it may be present in female meiotic cells as well [38]; in turn, TRIM11 is expressed in mice oocytes in meiotic prophase I and in ovaries of mice embryos [40], [41]. Another strong association is SNP rs2505115 (p = 1.83×10^−6^; see Table 1), ∼1 kb downstream of KIAA1462 and 200 kb upstream of MTPAP [19] (Figure 5B). Both KIAA1462 and MTPAP are highly expressed in mice oocytes in meiotic prophase I and in ovaries of mice embryos [40], [41].

None of the four signals (assigned to genes NUB1, UGCG, PDZK1 and KIAA1462) previously reported to be associated with sex-specific mean recombination rate in these same two population samples [19] meet genome-wide significance in our analysis of the data (nor did they in the original analysis [19]). Moreover, while two of the SNPs have low p-values (p = 6×10^−6^ for KIAA1462 and female mean rates; p = 2×10^−6^ for NUB1 and male mean rates), falling in the top three most strongly associated regions, two do not (p = 0.005 for PDZK1 and female mean rates; p = 0.014 for UGCG and male mean rates). We investigated the source of the discrepancy and concluded that two of the previous associations are likely spurious and due to errors in phenotype estimation (see Materials S1). In any case, since little is known about these genes, and they do not meet genome-wide significance, all four loci probably need further replication before they can be considered strong candidates.

As our third phenotype, we tested for loci associated with historical hotspot usage in a meta-analysis of the FHS and AGRE data. Since there is no evidence for a marked difference in historical hotspot usage for males and females in these data (see also [10], [20]), we only present results for this phenotype in the two sexes jointly. Given our sample sizes and assuming the causative SNP is present on the array or well tagged, we expect to have over 99% power to detect an allele at 20% frequency with a ∼4% effect on hotpot usage (see Supplementary Figure 12 in Materials S1). The one SNP to meet genome-wide significance is near PRDM9 (p = 1.3×10^−8^). This finding confirms the strong effect of this locus in two additional population samples. In the HUTT, variation in the zinc finger of PRDM9 alone appears to explain most (but perhaps not all) of the estimated narrow sense heritability in this trait: after regressing the genotype of PRDM9 for 317 of the 326 parents with typed PRDM9 alleles, h^2^ = 0.059 (p = 0.06).

**Figure 6:**
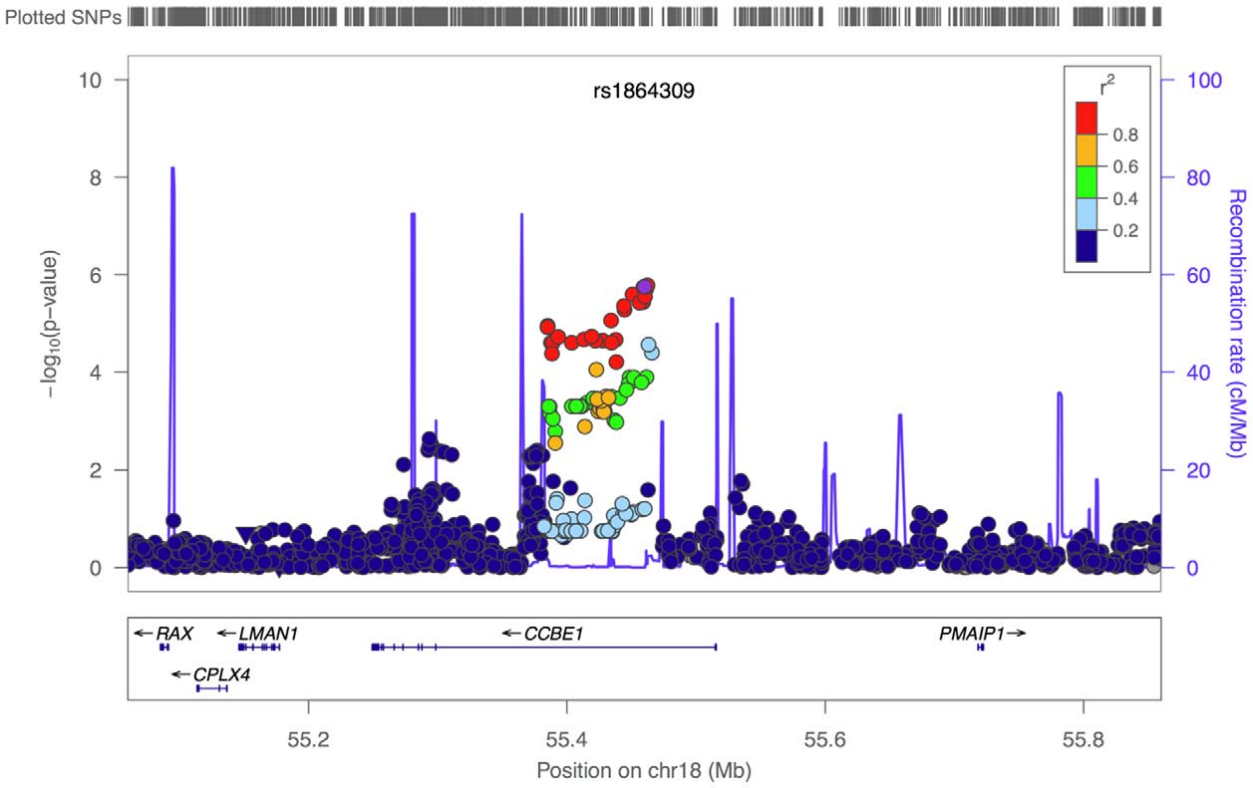
A close up of the association signal at a new candidate region for hotspot usage. The plot was generated using the software LocusZoom [51], as described in the legend of Figure 4.

Given the large role of PRDM9 on historical hotspot usage, we regressed out the genotype of the three most strongly associated SNPs at PRDM9 and reran the test for association. The strongest associations have p-values < 10^−5^ but do not reach genome-wide significance (Table 1) (the same is true when analyzing sex-specific phenotypes). Among top signals is a region of high linkage disequilibrium in an intron of CCBE1 (p = 5.75×10^−6^) (Figure 6), a gene expressed in mice oocytes in meiotic prophase I and in ovaries of embryos [40], [41] and more tentatively in human testes and mouse spermatocytes [38]. Moreover, two of the SNPs in high LD with the top SNP are reported to be trans eQTLs for PHF5A, a splicing factor subunit that is expressed in mouse spermatocytes from leptotene through pachytene [42].

We further examined whether any SNPs strongly associated with either historical hotspot usage or mean rate were also strongly associated with the other phenotype. That was the case for neither RNF212 nor PRDM9. More generally, we found no broader evidence of SNPs strongly associated with one phenotype being enriched for an association with the other phenotype (results not shown). This finding provides further evidence that variation in mean recombination rate and hotspot usage have distinct sources.

### Assessing further support for the novel candidate regions

We considered whether the HUTT show an enrichment of low p-values for the 150 SNPs that were most strongly associated with a recombination phenotype in the meta-analysis of FHS and AGRE. There was no significant enrichment at the 1 or 5% level (results not shown). However, the HUTT sample is small so we only have high power to detect large effect sizes.

In addition, we asked whether the top SNPs from the meta-analysis were enriched for genes known to be involved in recombination in model organisms (using a list kindly provided to us by Neil Hunter). While we observed no significant enrichment of our association signals in this set of genes (see Materials S1), we did find intriguing signals at RTEL1, SENP1 and PIAS1 (see Supplementary Table 4 in Materials S1).

## Discussion

These results constitute the most comprehensive characterization of variation in human recombination phenotypes to date. They show that mean recombination rates in males and females and historical hotspot usage have significant components of additive genetic variance, and that the two phenotypes are not correlated, suggesting that the genetic map length and fine-scale positioning of events are separately determined [43], [44].

For three of the phenotypes, the narrow sense heritabilities were not significantly different from 0: Myers motif usage and centromere and telomere usage (including for more stringent definitions; see Materials S1). Although these findings may indicate that the phenotypes are indeed not heritable, given the imprecision of the phenotype measurements, they could also reflect a downwards bias in our estimates of heritability. In addition, Myers motif usage may be a poorly defined phenotype, as it relies on an estimate of the penetrance of the motif at individual hotspots, which may be unreliable [20], [21].

Focusing on the three phenotypes with significant heritabilities, we performed one of the first genome-wide association studies of recombination phenotypes. We found that the variance in historical hotspot usage appears to be largely due to one gene, PRDM9, which explains over half of the estimated heritability. Thus, somewhat surprisingly, this trait appears to have a simple genetic basis. The mean recombination rate in males also appears to be influenced by one locus of relatively large effect, RNF212. Beyond that, these data do not provide statistical support for large effect, common recombination modifiers in the two-thirds of the genome that should be well tagged by this set of markers [35], [36]. This is even more apparent for the female mean rate, in which there are no associations that meet genome-wide significance, when we would expect over 95% power to detect common variants with a 2 Morgans (i.e., ∼5%) effect on mean rates. Thus, although the female mean rate has a similar narrow sense heritability estimate to historical hotspot usage, it seems to be much more complex in its genetic basis. Moreover, there are clear environmental effects, as evidenced by the large variation among oocytes of the same female (e.g., [5]), and the effect of maternal age [9], [10]. Also of note, there are no strong associations to mean recombination rate when male and female rates are combined, indicating that the few loci of relatively large effect are sex-specific [19], a finding supported by the tiny additive genetic covariance between male and female rates.

While the genome-wide association study identified a set of promising candidate regions, these associations do not reach genome-wide significance, so their roles in modifying recombination phenotypes remains to be replicated. To date then, we know of three loci that clearly influence human recombination phenotypes: RNF212, Inversion 17q21.31 and PRDM9. The zinc finger domain of PRDM9 is unusually rapidly evolving among mammalian species, notably apes, and haplotype diversity differs markedly among human populations [8], [21], [22], [26], [27]. In turn, SNPs in RNF212 and Inversion 17q21.31 are somewhat unusually differentiated among populations [16], [18]. Whether it is a coincidence that the first three loci all show signs of rapid evolution awaits the discovery of additional genes that influence variation in human recombination. As that set grows, it will also be of interest to contrast the selection pressures on modifiers of the total genetic map length versus on modifers of local rates, as well as to examine whether the same loci shape recombination rate variation in humans and in other species.

## Materials and Methods

### Data sets

We focused on (potentially overlapping) nuclear families of two or more offspring, in which parents and children had been genotyped with genome-wide arrays. We analyzed three population samples of European ancestry, including 732 families in the Framingham Heart Study (FHS) [45], [46] with a median of 3 offspring; 444 families in an Autism Cohort (AGRE) [47], who had a median family size of 2; and 163 families from a founder population (HUTT) [48], who had a median family size of 4. Further details about the data sets are provided in Materials S1.

In the AGRE and FHS datasets, all individuals were typed with the Affymetrix GeneChip Mapping 500K Array set, as were most HUTT individuals. A minority of HUTT individuals were typed with two other Affymetrix arrays (Affymetrix® Genome-Wide Human SNP Array 5.0, Affymetrix® Genome-Wide Human SNP Array 6.0), and so for the analysis of that population sample, we took the intersection of SNPs on the three arrays. To infer crossovers, we focused on the autosomes, and applied a number of quality control filters to the data, based on the call rate, the Mendelian error rate, measures of identity-by-descent, and tests of Hardy-Weinberg disequilibrium; these steps are detailed in Materials S1. For the genome-wide association study, we also considered X-linked markers, and to all SNPs applied a minimum allele frequency cut-off of 5%.

### Crossover calls

We inferred the location of crossovers in the offspring by the method of Coop et al. (2008)[10] and by a new Hidden Markov Model that we developed (see Materials S1 for details). Results were highly concordant between the two methods, as detailed in Materials S1, so we proceeded with the results from the method of Coop et al. (2008). In addition to the comparison between methods, we checked the reliability of our crossover calls by comparison to previous studies; at the megabase scale, the genetic maps were highly similar between population samples, and by comparison to the map of Kong et al. (2002)[49] (see Materials S1).

### Five phenotypes

We estimated five recombination phenotypes for each parent, as listed in the main text and detailed in the Materials S1 and Materials S2. We estimated the narrow sense heritability of these phenotypes in the HUTT sample by using a variance component, maximum-likelihood method [50] (see Materials S1).

### Genome-wide association study

We conducted a genome-wide association study of mean recombination rate in males and in females as well as of historical hotspot usage in the two sexes. First, we regressed the recombination phenotype on the family size (coded as a categorical variable), because we were concerned that the performance of our crossover calling method may depend weakly on the number of offspring in a family. In FHS and AGRE, where there may be population substructure in the phenotype distribution by chance or true population differences in recombination phenotypes, we considered only parents with European ancestry and regressed out ancestry-informative PCs. We then ran a linear regression of the residuals on the genotypes. The association study in the HUTT was conducted by the program GTAM, which tests for an association using an additive model, while accounting for the relatedness in the Hutterite pedigree [34]. For more details, see Materials S1.

## Supporting Information

Materials S1 **A description of the analyses summarized in the** **Materials and Methods** **section, including supplemanty figures and tables.**
(DOC)

Materials S2 **The table contains the autosomal recombination events detected in FHS, AGRE, and HUTT datasets (first column). Each row is a recombination event. The second and third columns give the sex and chromosome that the recombination event occurred in. The fourth and fifth columns give the positions that bracket the interval in which the recombination event was observed within (positions are mapped to hg18). For the latest version of the data set, please check the websites of G. Coop or M. Przeworski.**
(XLSX)
