# Supplementary material for: Variation in Human Recombination Rates and Its Genetic Determinants
Source: PLoS One. 2011 Jun 17;6(6):e20321. doi: 10.1371/journal.pone.0020321 (PMC3117798; doi:10.1371/journal.pone.0020321)
Supplement: Materials S1 — A description of the analyses summarized in the Materials and Methods section, including supplemanty figures and tables. (DOC) [file pone.0020321.s001.doc]

**SUPPLEMENTARY MATERIALS** for

Variation in human recombination rates and its genetic determinants

Adi Fledel-Alon^,*, Ellen Miranda Leffler^,*, Yongtao Guan^,&, Matthew Stephens^,&, Graham Coop^!,+, $^ and Molly Przeworski^,%, #,+, =

^*^ contributed equally

^+^ co-supervised this project

^ Dept. of Human Genetics, University of Chicago, Chicago IL

^&^ Dept. of Statistics, University of Chicago, Chicago IL

^!^ Dept. Of Evolution and Ecology and Center for Population Biology, University of California Davis, Davis CA

% Dept. of Ecology and Evolution, University of Chicago, Chicago IL

^#^ Howard Hughes Medical Institute

= To whom correspondence should be addressed: [gmcoop@ucdavis.edu](mailto:gmcoop@ucdavis.edu), [mfp@uchicago.edu](mailto:mfp@uchicago.edu)

We tested for an association between autosomal recombination phenotypes and single nucleotide polymorphisms (SNPs) in three sets of pedigrees, from the Framingham Heart Study (FHS), the Autism Genetic Resource Exchange (AGRE) and the Hutterite population (HUTT). To this end, we ran a number of quality controls on the genotype data, inferred the location of crossover events, estimated recombination phenotypes and ran a genome-wide association. We also analysed FHS and AGRE results jointly, in a meta-analysis. These steps are detailed in what follows.

**Quality control filters for FHS samples.** The FHS made publicly available genotype data for 9,237 individuals of European ancestry [1,2]. We focused on the subset of individuals that belonged to families with two or more offspring (since trios cannot be used to identify crossover events). This left us with 3,695 individuals, who belong to 783 (overlapping) nuclear families. These individuals had all been typed with the Affymetrix GeneChip Mapping 500k Array Set. The genotypes were called using the BRLMM software (<http://www.affymetrix.com/support/technical/whitepapers/brlmm_whitepaper.pdf>); genotypes with confidence scores above 0.5 were not provided. We downloaded the genotype and pedigree data from dbGAP ([http://www.ncbi.nlm.nih.gov/projects/gap/cgi-bin/study.cgi?study_id=phs000007.v6.p3](http://www.ncbi.nlm.nih.gov/projects/gap/cgi--bin/study.cgi?study_id=phs000007.v6.p3)).

The 500K Affymetrix array interrogates variation at 500,568 single nucleotide polymorphisms (SNPs). We focused on the 487,014 autosomal and 9,931 X-linked SNPs that map to known positions in the human genome build 36 (<http://genome.ucsc.edu/>). We then implemented a number of quality control steps, using the software PLINK (version 1.06,

<http://pngu.mgh.harvard.edu/purcell/plink/>; [3]): (i) We excluded 26 individuals with a call rate below 90%. (ii) We calculated the number of Mendelian errors per individual and excluded those in the top 0.03%-tile. In addition, using patterns of identity by descent, we identified six pairs of monozygotic twins. We retained one twin at random and excluded the two families for which, as a result, we only had data from a single child. (iib) Using principal components, we identified and excluded one father with probable East Asian ancestry. (iic) Noticing a weak correlation between recombination phenotypes and parental missing data (not shown), we further excluded the 28 parents with greater than 4% missing data. (iii) Next, we considered the call rates and Mendelian error rates per SNP. Specifically, we removed the SNPs that had the lowest 5%-tile of call rates as well as SNPs with the highest 5%-tile of Mendelian errors, for a total of 32,080 SNPs. The median call rate for the remaining SNPs is 99.5% (mean 98.8%). At the end of this process, we had genotype information for 454,934 SNPs, of which 428,654 were polymorphic in our sample (median distance of 3.56 kbs), which we used to infer recombination events. (iv) Finally, we sought to impose a genotype level quality control step, by converting calls with the lowest 1%-tile of confidence scores to missing data. This criterion turned out to be less stringent than the one already imposed by default by the BRLMM software, so we proceeded with all the available calls.

For the genome-wide association study (GWAS), which is more sensitive to genotyping error than our recombination calling method, we imposed a further filter on the SNP data, excluding SNPs for which a test of Hardy-Weinberg Equilibrium (on the founders of the pedigree) yielded p < 0.001. In addition, we only considered SNPs for which the minor allele frequency (among individuals for which we had phenotypes) was greater than 0.05. We applied the same percentage thresholds and filters to SNPs on the X chromosome. This left us with 343,305 autosomal markers and 6,595 X-linked markers to test for an association.

**Quality control filters for AGRE samples.** The data were made available by the AGRE project [4] and consisted of 524 families of two or more offspring. The same filters were applied as to the FHS data, with a few exceptions noted below. Specifically: (i) Using patterns of identity by descent, we identified one set of quadruplets and 19 pairs of monozygotic twins. We retained eight families where we could exclude one of each twin chosen at random, and excluded the 12 families for which, as a result, there were fewer than two children. (ii) Using the X chromosome data to verify the sex of the individual, we identified three couples in which the sex labels had been switched and corrected them. (iii) We ran the program smartPCA [5] on the parents along with HapMap CEU, YRI, CHB, and JPT individuals, and retained only those parents who clustered with the CEU (which was largely consistent with a self-reported ancestry of «white, non-Hispanic»). (iv) Next, we considered call rates and Mendelian error rates per SNP. We did not remove any additional SNPs based on these criteria because the original dataset had a much higher call rate in comparison to the FHS data, and SNPs with more than 20 Mendelian errors had already been removed.

For the genome-wide association study (GWAS), we imposed a further filter on the SNP data, excluding SNPs for which a test of Hardy-Weinberg Equilibrium (on the founders in the pedigrees) yielded p < 0.001. In addition, we only considered SNPs for which the minor allele frequency (among individuals for which we had phenotypes) was greater than 0.05. This left us with 336,006 autosomal markers and 6,842 X-linked markers to test for an association.

**The Hutterite population sample.** We also analyzed genotype data from a population sample of Hutterites, a founder population of European ancestry now living in North America (cf. [6]). All the individuals in the sample are part of a single well-documented 13 generation pedigree, which can be traced back to 64 founders [7]. For the purposes of our analysis, we broke the pedigree up into overlapping nuclear families in which both parents and at least two children had been genotyped, i.e., 173 families with a median number of 4 genotyped children (mean 4.7). A mixture of three genotyping platforms were used: 537 individuals were typed by the Affymetrix GeneChip Mapping 500K Array set (most of which had been studied in [8,9]), 136 by the the Affymetrix® Genome-Wide Human SNP Array 5.0 and 386 by the Affymetrix® Genome-Wide Human SNP Array 6.0. The genotyping and genotyping calling was performed by the Vanderbilt Functional Genomics shared resource (FGSR), using the BRLMM algorithm (<http://www.affymetrix.com/support/technical/whitepapers/brlmm_whitepaper.pdf>), the BRLMM-p algorithm (<http://www.affymetrix.com/support/technical/whitepapers/brlmmp_whitepaper.pdf>) and Birdseed V2

(<http://www.broadinstitute.org/mpg/birdsuite/birdseed.html>) for the three platforms, respectively.

For these data, the quality control steps were as follows: (i) We excluded the one individual with a lower than 90% call rate. At this point, we took the intersection of SNPs on the three arrays, which left us with 404,864 markers. Of these, 350,259 were polymorphic in the sample. (ii) Examining patterns of identity by descent, we identified a pair of monozygotic twins, of which we retained only one individual. Using the pedigree information, we also found three mislabelled individuals, five misidentified relationships, and three individuals whose genotype data did not match the sex, leading us to exclude three families and eight children from six additional families. (iii) We then excluded the four individuals with the highest 0.03%-tile of Mendelian errors. (iv) We removed the SNPs that had the lowest 5%-tile of call rates and the highest 5%-tile of Mendelian errors. At the end of this process, we had genotype information for 372,163 SNPs, which were used to call crossover events. (v) Considering each platform separately, we turned the genotypes with the lowest 1%-tile of confidence scores into missing data. For the 500K array, this criterion had already been imposed by the Vanderbilt genotyping center. For the other two arrays, our criterion was more stringent, leading us to convert additional genotypes to missing data.

For the genome-wide association study, we considered only the subset of markers with minor allele frequency greater than 0.05 (among individuals with recombination phenotypes), i.e., 298,099 markers. We did not impose a Hardy-Weinberg Equilibrium cut-off because the relatedness among the Hutterites leads to a highly skewed distribution of p-values (results not shown).

**Inferred crossover events.** In the FHS sample, we inferred crossover events on the autosomes using both a previously described heuristic method [9] and a new Hidden Markov Model (HMM) method (see below for a description). Our motivation in developing the HMM was a concern that our heuristic method, which is essentially a parsimony approach, would not perform reliably on families with relatively few children. We found, however, that the two methods yielded highly concordant results (see below). Thus, in the main paper, we report results for the Coop et al. method, variants of which have been implemented in subsequent papers [10,11].

We used the Coop et al. method as previously described [9], requiring K=5 consecutive informative markers in order to call a crossover (in order to minimize the effects of genotyping errors) and using additional markers to narrow down the interval when possible (see [9] for details). In FHS, there were three families (with 6-7 children) that included one individual with an excess of crossovers on one of the chromosomes in the maternal or paternal transmission; we excluded the three individuals. We also excluded one family with an excess of crossovers on chromosome 22, likely due to poor genotyping quality. The final sample consisted in 732 mothers and 732 fathers in 745 families, with a median of 3 children (and a mean of 2.83). In total, we inferred the occurrence of 143,281 autosomal recombination events from 2077 male and 2083 female meioses. The median interval size within which we could delimit the events is 80.1 kb, with 24% of crossovers called in an interval smaller than 30 kb (see Supplementary Figure 1). In AGRE, the post-QC sample included 422 fathers and 417 mothers in 444 families, with a median of 2 children per family (mean of 2.24). We inferred a total of 66,380 autosomal recombination events from 947 male and 934 female meioses. The median interval size within which we could delimit the events is 104.08 kb, with 17% of crossovers called in an interval smaller than 30 kb. After removing one family that was a clear outlier in terms of the number of crossovers, the Hutterite sample consisted in 163 mothers and 163 fathers in 163 families, with a median of 4 children (mean 4.62). We inferred 49,326 crossover events on the autosomes from 757 male and 757 female meioses. The median interval size within which we could delimit the events is 99.54 kb, with 19% called in an interval smaller than 30 kb.

**Supplementary Figure 1**: The distributions of interval sizes into which crossover events are resolved.

In the course of this analysis, we found an error in the implementation of crossover calling in [9] (but not [12]): when inferring crossovers in families with an even number of children, the crossover events that occurred in the same genomic interval in exactly half of the offspring (e.g., 2 out of 4) were erroneously assigned to all the offspring. This led to a slight increase in recombination rates in families with four children, but had no significant effect on our previous analysis. Here, we assigned such events to half the offspring, and chose which half at random (since our method does not allow us to identify unambiguously which half are truly recombinants). This problem occurred in ~0.9% of calls in the FHS data, ~0.01% in the AGRE data and ~1.4% in the Hutterite data.

We assessed the reliability of our crossover calls in a number of ways, including by comparison to those obtained by an HMM method (see below). In particular, we compared the recombination rates that we inferred in FHS, AGRE and HUTT to those obtained by Kong et al. (2002) [13] based on more meioses but many fewer markers. Even though the Kong et al. genetic map relies on an estimate of crossover interference whereas we do not (given the high density of our markers), the concordance is excellent, as shown in Supplementary Figure 2 for exemplar chromosomes 1 and 2. The agreement provides further confidence that our approach to calling crossovers is reliable and indicates that, at this scale, the three populations have highly similar genetic maps.

**Supplementary Figure 2**: The concordance of genetic maps, shown for chromosomes 1 and 2.


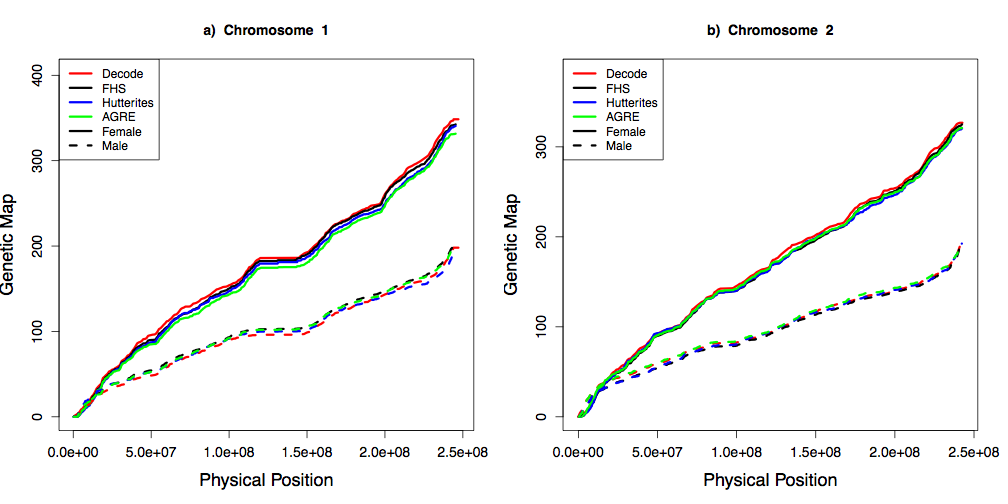


Male and female cumulative genetic distances inferred from FHS and Hutterite data, for chromosomes 1 and 2. The black lines are for the FHS, the green lines for AGRE, the blue lines are for the Hutterites, the red lines for the Kong et al. genetic map; dashed is in males and solid in females.

**Recombination phenotypes.** We estimated five recombination phenotypes for each parent in our sample, provided in Text S2. We note that estimates of these phenotypes from pedigrees are necessarily accompanied by substantial error, because they rely on a small number of children and for phenotypes (2)-(5), on a subset of the crossovers.

(1) The genome-wide recombination rate per individual, i.e., the mean number of crossovers per individual (averaged over their offspring). Variation in this phenotype is shown in Supplementary Figure 3.

**Supplementary Figure 3**: Variation in the mean recombination rate among individuals.


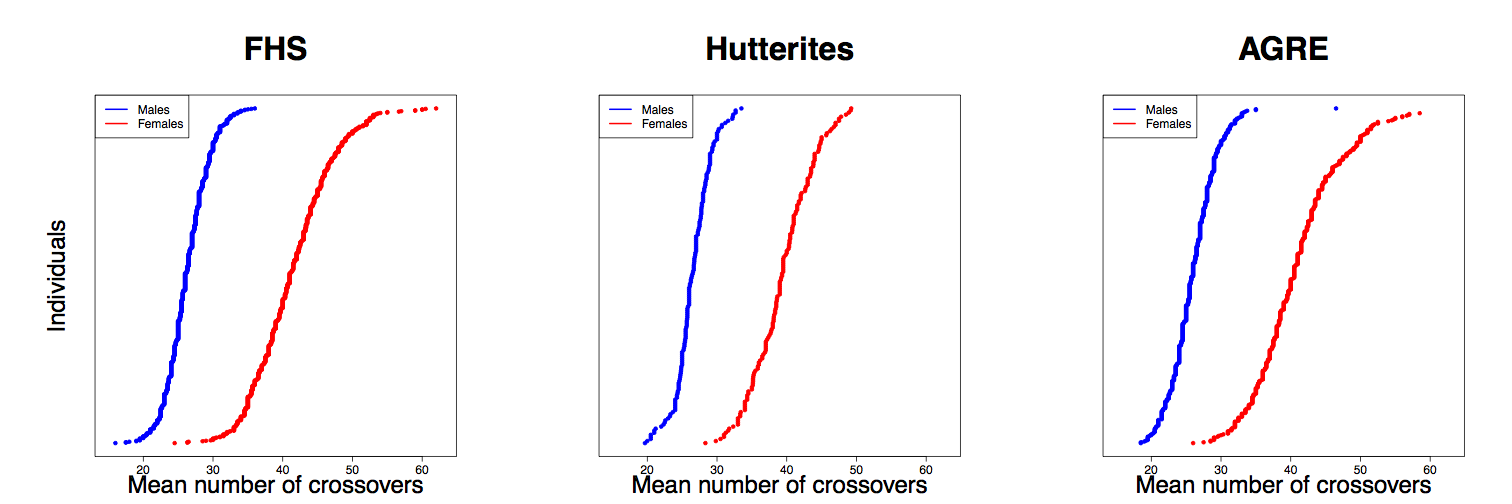


(2) The fraction of crossovers that occur in the 20% most telomeric regions of all autosomes. For phenotypes (2) and (3), these regions were defined using the table «gap» in the UCSC genome browser; the locations of crossovers were determined using the mid-point of the interval in which a crossover was localized. Variation in this phenotype is shown in Supplementary Figure 4.

**Supplementary Figure 4**: Variation in telomere use among individuals.


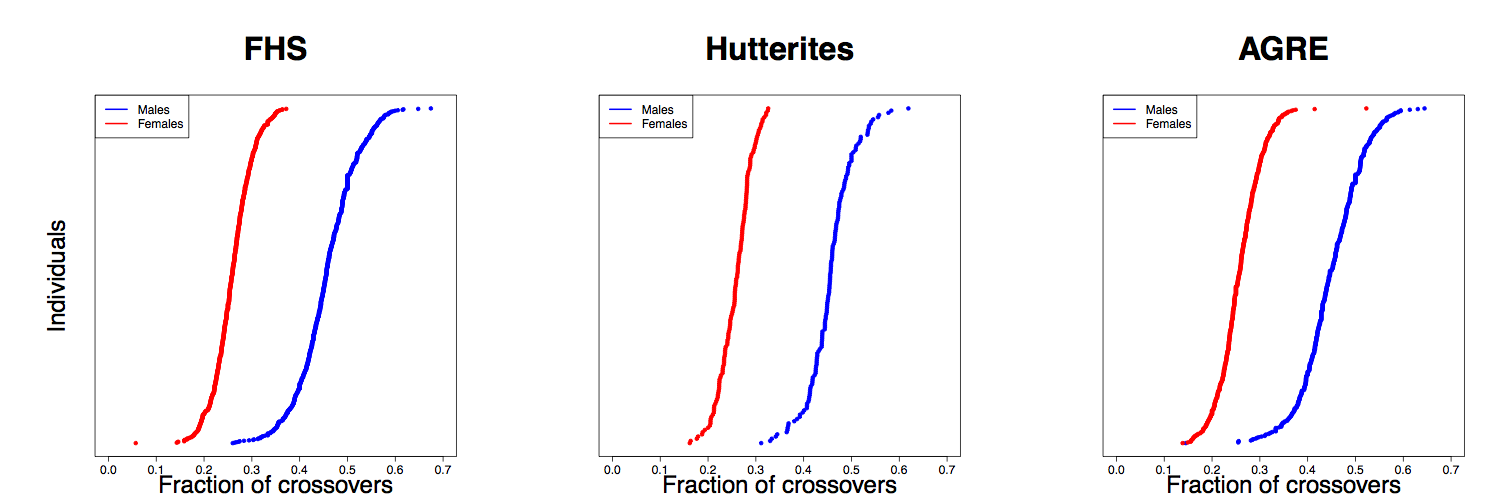


(3) The fraction of crossovers that occur in the 20% most centromeric regions of all autosomes. Variation in this phenotype is shown in Supplementary Figure 5.

**Supplementary Figure 5**: Variation in centromere use among individuals.


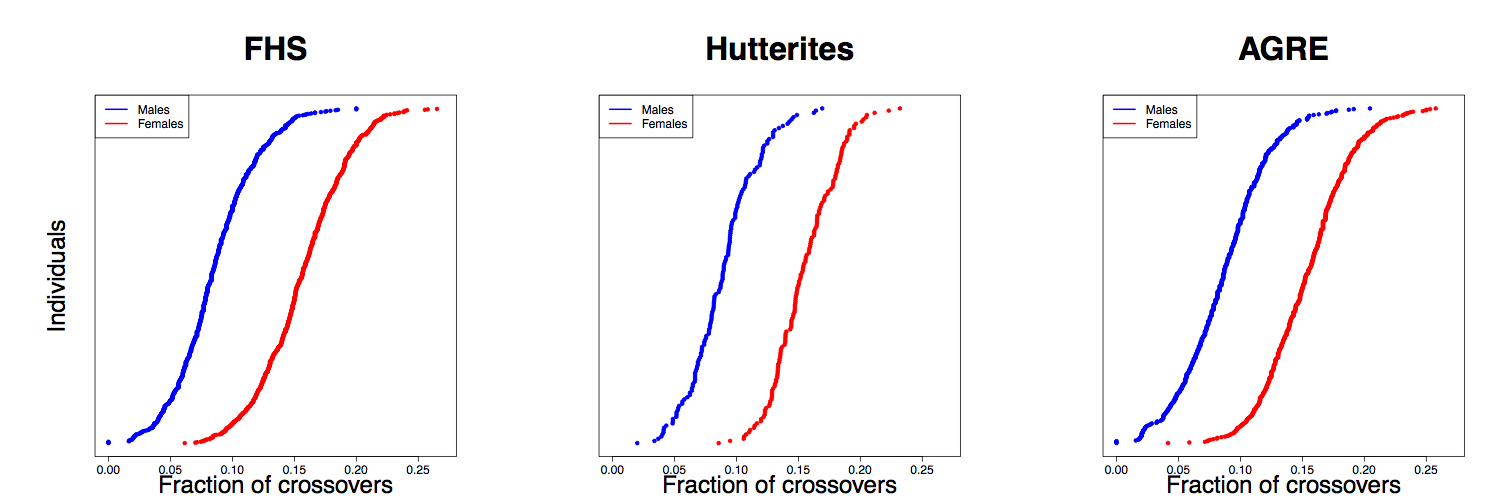


(4) The genome-wide «historical hotspot usage» per individual. This phenotype was defined and estimated as in [9]. Namely, we considered hotspot usage to be the fraction of well-defined crossover events that overlap a recombination hotspot inferred from linkage disequilibrium (LD) data, genome-wide. For each individual, we estimated this fraction, α, using a maximum likelihood approach, considering well-defined crossovers to be those delimited to within 30 kb and using the historical hotspots inferred by Myers et al. (2005) [14]. This approach corrects for the possibility of overlap by chance [9]. As a sanity check, we also considered the proportion of overlap per individual (which does not correct for overlap by chance). This second phenotype was highly correlated with the α estimates (results not shown). Variation in this phenotype is shown in Supplementary Figure 6.

**Supplementary Figure 6**: Variation in hotspot use among individuals.


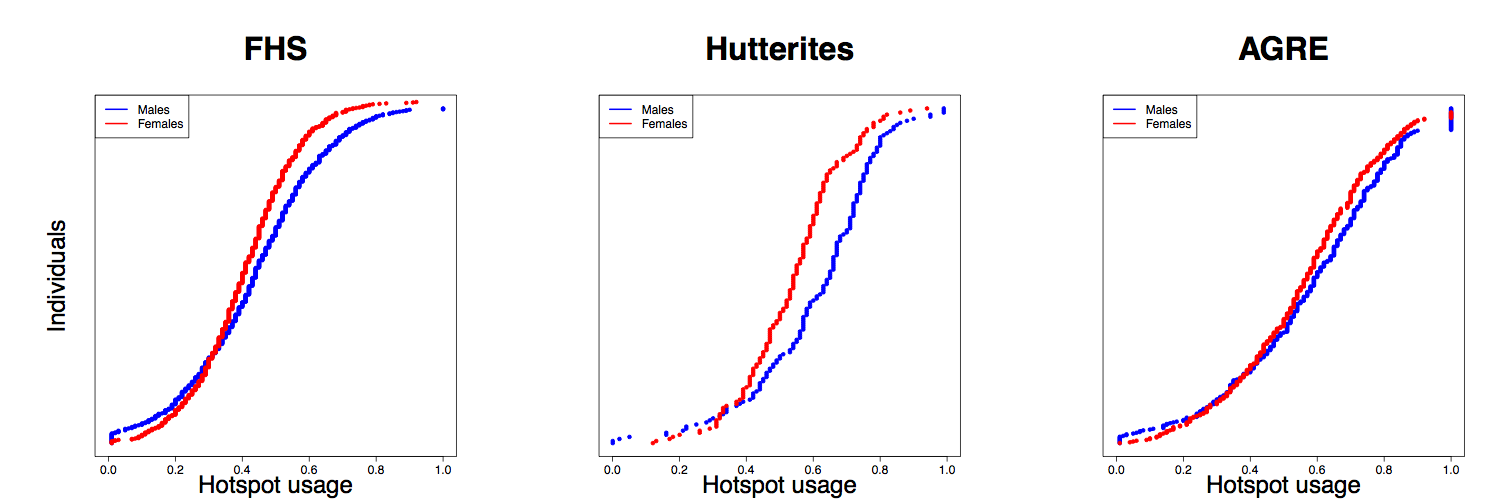


As can be seen, the mean level of historical hotspot usage differs across the three studies. Within studies, there is a weak negative correlation between the fraction of missing data and the hotspot usage phenotype (leading us to treat the fraction of a missing data as a covariate in our association studies of FHS and AGRE). Since the FHS dataset had both the highest rate of missing data and the lowest historical hotspot usage of the three studies, the differences among studies could potentially be due to the levels of missing data. We did not pursue this issue further, as for the purposes of the genome-wide association study, we are interested in the variation within each study rather than differences among them. However, this observation suggests that the historical hotspot usage phenotype, while a useful phenotype for mapping studies (as shown by the association to PRDM9 [8]), is an imperfect proxy for the underlying biological phenotype.

(5) We estimated an additional phenotype, referred to as «Myers motif hotspot usage». For a set of 22,699 hotspots that are identified from patterns of LD and well-localized, Myers et al. (2008) [15] estimated the probability that a given hotspot was caused by the degenerate 13-mer motif (since shown to be bound by PRDM9 [8,16]). These «motif probabilities» were kindly provided to us by S. Myers (University of Oxford). For each individual, we estimated the fraction of crossover events in hotspots that were caused by the Myers et al. motif. Specifically, we considered crossovers that are delimited to an interval of < 30kb and overlap at least one hotspot identified from LD. For each crossover, we then averaged the motif probabilities across the LD-based hotspots that the interval overlaps. This procedure provides an approximate estimate of the probability that, given that a crossover occured in a LD-based hotspot, it was caused by the motif. For each parent, we averaged these estimates over their crossovers, ignoring events that are not refined to within 30kb or that do not overlap an LD-based hotspot. A more sophisticated approach would also take into account the length of the interval within which each crossover is delimited, and the number of hotspots that each crossover interval overlaps. However, our simple phenotype is highly correlated with these more complex approximations (results not shown), and we therefore chose to use the simplest calculation as the phenotype. Variation in this phenotype is shown in Supplementary Figure 7.

**Supplementary Figure 7**: Variation in Myers motif use among individuals.


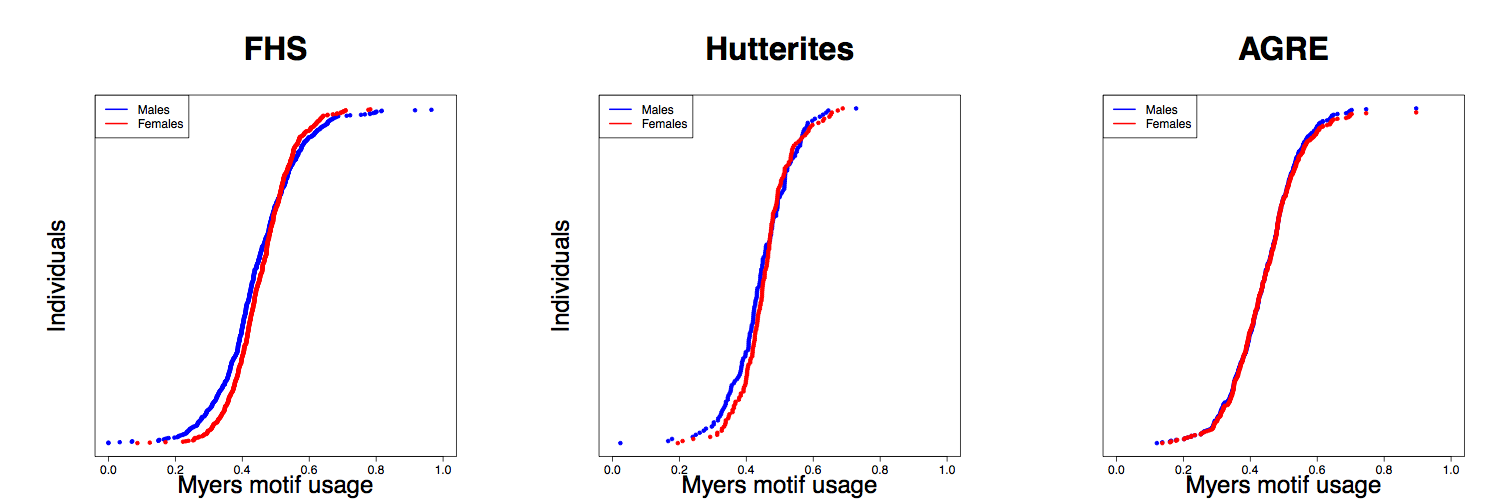


We then considered the correlation among these five phenotypes (Supplementary Figure 8). Strikingly, the only significant correlation seen in all three population samples is between the fraction of crossovers that occur in the 20% most telomeric regions and 20% most centromeric regions (p<10^-5^). In no case was the mean rate and hotspot usage significant at the 5% level.

**Supplementary Figure 8**: The correlation among recombination phenotypes, in the AGRE, FHS and HUTT data.


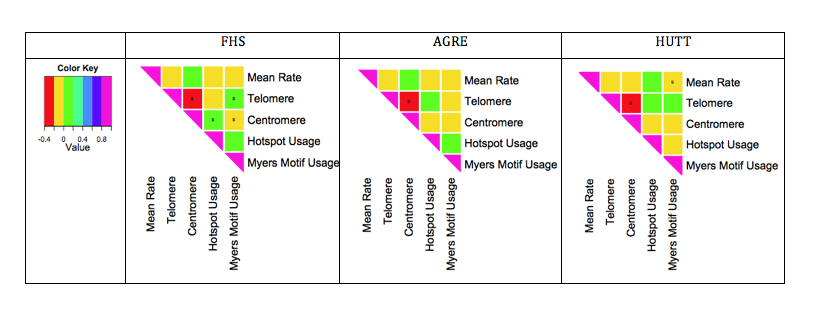


**Heritability estimates from the Hutterites.** We took advantage of the fact that the Hutterite parents are related within a 3,671-person pedigree in order to estimate the heritability of our five phenotypes using a variance component, maximum-likelihood method [17]. For this application, we considered only environmental and autosomal additive variance components (i.e., we did not estimate dominance variance component and other higher order variance components)*.* The method estimates both variance components and then uses them to estimate the narrow-sense heritability as *h^2^* = (1+*f*)*V_A_*/*V_T_*, where *f*  is the average inbreeding coefficient of the population and *V_A_* and *V_T_* are the additive and total variances, respectively.

**Supplementary Table 1**: Estimated heritabilities of the five recombination phenotypes, in the Hutterite sample

|  | **h^2^** | **SE** | **p-value** |
| --- | --- | --- | --- |
| Recombination rate (combined) | 0.113 | 0.088 | 0.037 |
| Male recombination rate | 0.142 | 0.135 | 0.078 |
| Female recombination rate | 0.253 | 0.186 | 0.015 |
|  |  |  |  |
| Historical hotspot usage (combined) | 0.227 | 0.106 | 0.011 |
| Male historical hotspot usage | 0.049 | 0.102 | 0.159 |
| Female historical hotspot usage | 0.000 | 0.090 | 0.276 |
|  |  |  |  |
| Myers motif hotspot usage (combined) | 0.000 | 0.042 | 0.709 |
| Male Myers motif hotspot usage | 0.000 | 0.074 | 0.962 |
| Female Myers motif hotspot usage | 0.000 | 0.090 | 0.534 |
|  |  |  |  |
| Centromere usage (combined) | 0.000 | 0.042 | 0.367 |
| Male centromere usage | 0.000 | 0.074 | 0.768 |
| Female centromere usage | 0.000 | 0.090 | 0.767 |
|  |  |  |  |
| Telomere usage (combined) | 0.000 | 0.042 | 0.955 |
| Male telomere usage | 0.000 | 0.074 | 0.709 |
| Female telomere usage | 0.000 | 0.090 | 0.598 |

The narrow sense heritabilities estimated for our five phenotypes are shown in Supplementary Table 1. Significance was assessed by permuting phenotypes over individuals within family size (pooling larger families to have at least 15 individuals in each group) and then estimating the heritability. From the 1000 permutations, the p-value was estimated as the number of heritability estimates equal to or greater than the observed heritability. As can be seen, hotspot usage shows a substantial heritability, in close agreement to the value obtained using a somewhat smaller sample [9]. Both female and to a lesser extent male mean recombination rate also show significant heritability. In contrast, the three other phenotypes do not have significant heritability. This is also true when we used different fractions of the chromosome arm (1/10, 1/20, 1/50 and 1/100) as our definition of telomere or centromere usage (results not shown).

**The genome-wide association study  in FHS and AGRE.** We conducted a genome-wide association study of the mean recombination rate and hotspot usage, in males, in females and in the two sexes jointly (i.e., a total of 6 associations). First, we regressed the recombination phenotype on the family size (coded as a categorical variable), because we were concerned that the performance of our crossover calling method may depend (weakly) on the number of offspring in a family. We also regressed out the first two principle components, which were informative about ancestry (see above). For hotspot usage, which was weakly correlated with the proportion of missing data, we additionally regressed out the proportion of missing SNPs. When considering males and females jointly, we standardized our phenotype within each sex (i.e., we subtracted off the mean and divided through by the standard deviation within each sex), because the female genetic map lengths are ~60% longer than that of males and more variable across individuals [18]; this approach implicitly assumes that alleles will have the same directional effect in the two sexes. We then ran a linear regression of the residuals on the genotypes. In addition, we ran a linear regression of quantile-normalized residuals on the genotypes; the set of top candidates remained highly similar (results not shown).

To estimate a threshold for genome-wide significance, we permuted phenotypes over individuals, assigning the phenotype at random to a parent with the same number of offspring (for this purpose, we pooled parents with 6 or more offspring in the FHS, to ensure that there would be sufficient numbers of each family size). The resulting thresholds were close to those obtained from a simple Bonferroni correction (not shown), so we used p < 2x10^-7^ as an approximate cut-off (i.e., 0.05 divided by the number of markers). These analyses were done using the software PLINK or our own scripts, written in R (http://www.r-project.org/).

Finally, we performed a fixed-effects meta-analysis of the association test results from the FHS and AGRE samples, using PLINK. There were 308,869 autosomal and 5,796 X-linked SNPs that had been tested in both samples and so were included in the meta-analysis. The top signals for each association study are shown in Supplementary Table 2.

**Testing for associations in the Hutterites.**  We focused on the top 0.048% (i.e., 150 SNPs) of significant associations for each test (using the PLINK results), and examined whether they replicated in the population sample of Hutterite individuals. To this end, we used the program GTAM [19], which tests for an association using an additive model, while accounting for the Hutterite pedigree.

We estimated the proportion of the heritability in male mean rate explained by RNF212 by regressing out the top three SNPs at RNF212 from the male mean rate phenotypes in the Hutterites and estimating heritability again. In turn, we performed the same estimation for historical hotspot usage by using the zinc finger genotypes determined in Baudat et al. [8] for 317 of 326 individuals used in the association study.

**Supplementary Table 2**: Strongest associations in meta-analysis of FHS and AGRE

| CHR | SNP | Position (bp) | Gene | Left Gene | Right Gene | MAF (FHS) | MAF (AGRE) | P | FHS | AGRE | HUTT |
| --- | --- | --- | --- | --- | --- | --- | --- | --- | --- | --- | --- |
| Male Mean Rate | | | | | | | | | | | |
| 4 * | rs11939380 | 1076871 | RNF212 | FGFRL1 | SPON2 | 0.320 | 0.330 | 7.10x10^-16^ | 4.98x10^-13^ | 5.98x10^-4^ | 4.37x10^-3^ |
| 5 | rs17542943 | 17123705 | NA | MYO10 | LOC285696 | 0.113 | 0.114 | 7.60x10^-7^ | 5.79x10^-5^ | 4.69x10^-3^ | 5.52x10^-1^ |
| 7 * | rs11764733 | 150707594 | NA | NUB1 | WDR86 | 0.395 | 0.396 | 1.75x10^-6^ | 1.08x10^-4^ | 5.76x10^-3^ | 6.19x10^-1^ |
| 1 | rs4424511 | 207383253 | NA | PLXNA2 | LOC642587 | 0.485 | 0.481 | 5.75x10^-6^ | 3.02x10^-3^ | 2.85x10^-4^ | 4.34x10^-1^ |
| 19 | rs17775194 | 39965539 | NA | ZNF599 | ZNF30 | 0.150 | 0.154 | 1.40x10^-5^ | 6.06x10^-5^ | 6.61x10^-2^ | 4.82x10^-1^ |
| 2 | rs2580823 | 232514563 | NA | NPPC | DIS3L2 | 0.304 | 0.329 | 1.59x10^-5^ | 2.31x10^-5^ | 1.55x10^-1^ | 5.17x10^-1^ |
| 14 | rs1187590 | 54122960 | SAMD4A | CGRRF1 | SAMD4A | 0.104 | 0.108 | 1.62x10^-5^ | 5.13x10^-4^ | 1.13x10^-2^ | NA |
| 21 | rs17738942 | 34426411 | MRPS6 | SLC5A3 | C21orf82 | 0.065 | 0.062 | 1.70x10^-5^ | 3.47x10^-3^ | 7.69x10^-4^ | 9.12x10^-1^ |
| 17 | rs1607987 | 9702391 | GLP2R | DHRS7C | RCVRN | 0.118 | 0.103 | 2.02x10^-5^ | 2.50x10^-4^ | 3.17x10^-2^ | 3.66x10^-1^ |
| 4 | rs11936376 | 149442733 | NR3C2 | ARHGAP10 | DCLK2 | 0.209 | 0.212 | 4.53x10^-5^ | 4.50x10^-3^ | 2.36x10^-3^ | 5.32x10^-1^ |
| 10 | rs9415799 | 66652411 | NA | ANXA2P3 | CTNNA3 | 0.085 | 0.102 | 4.78x10^-5^ | 1.40x10^-2^ | 5.40x10^-4^ | 4.25x10^-1^ |
| 17 | rs1062683 | 3786434 | ATP2A3 | P2RX1 | ZZEF1 | 0.191 | 0.173 | 4.95x10^-5^ | 3.08x10^-4^ | 5.29x10^-2^ | 5.07x10^-2^ |
| 7 | rs4729116 | 93817535 | NA | BET1 | COL1A2 | 0.110 | 0.113 | 5.30x10^-5^ | 2.11x10^-2^ | 3.32x10^-4^ | 3.64x10^-1^ |
| 8 | rs2139454 | 50421664 | NA | C8orf22 | SNTG1 | 0.381 | 0.357 | 5.49x10^-5^ | 8.63x10^-3^ | 1.25x10^-3^ | 7.97x10^-1^ |
| 15 | rs7174629 | 99201112 | NA | ASB7 | ALDH1A3 | 0.426 | 0.425 | 5.50x10^-5^ | 1.40x10^-3^ | 1.43x10^-2^ | 1.15x10^-1^ |
| 2 | rs17363477 | 9848545 | NA | YWHAQ | TAF1B | 0.178 | 0.203 | 5.67x10^-5^ | 1.13x10^-4^ | 1.52x10^-1^ | 7.74x10^-1^ |
| 22 | rs5768793 | 45241983 | CELSR1 | TRMU | GRAMD4 | 0.420 | 0.411 | 6.00x10^-5^ | 2.34x10^-4^ | 8.66x10^-2^ | 6.63x10^-1^ |
| 12 | rs12581737 | 61411736 | PPM1H | MIRLET7I | AVPR1A | 0.172 | 0.182 | 7.10x10^-5^ | 2.60x10^-2^ | 2.11x10^-4^ | 9.55x10^-1^ |
| 3 | rs7635068 | 188814284 | NA | RTP4 | SST | 0.181 | 0.179 | 7.93x10^-5^ | 3.79x10^-4^ | 7.96x10^-2^ | 3.03x10^-2^ |
| 5 | rs2042003 | 93793121 | C5orf36 | POU5F2 | C5orf36 | 0.340 | 0.323 | 8.37x10^-5^ | 5.63x10^-2^ | 4.50x10^-5^ | 7.89x10^-1^ |
| 12 | rs278998 | 25658437 | IFLTD1 | IFLTD1 | RASSF8 | 0.288 | 0.256 | 8.75x10^-5^ | 1.18x10^-5^ | 5.72x10^-1^ | 6.20x10^-2^ |
| Female Mean Rate | | | | | | | | | | | |
| 9 | rs10985535 | 123918003 | NA | TTLL11 | NDUFA8 | 0.074 | 0.075 | 8.27x10^-7^ | 2.42x10^-5^ | 1.17x10^-2^ | NA |
| 1 | rs564636 | 226549992 | OBSCN | C1orf69 | TRIM11 | 0.325 | 0.298 | 1.39x10^-6^ | 1.47x10^-5^ | 2.98x10^-2^ | 7.88x10^-1^ |
| 10 * | rs2505115 | 30438797 | NA | KIAA1462 | MTPAP | 0.136 | 0.134 | 1.83x10^-6^ | 3.10x10^-4^ | 1.85x10^-3^ | 5.08x10^-1^ |
| 2 | rs7598320 | 123148391 | NA | TSN | CNTNAP5 | 0.166 | 0.136 | 3.06x10^-6^ | 1.73x10^-4^ | 6.31x10^-3^ | 3.99x10^-2^ |
| 15 | rs12441966 | 51028519 | NA | ONECUT1 | WDR72 | 0.253 | 0.276 | 3.62x10^-6^ | 2.10x10^-4^ | 6.11x10^-3^ | 7.80x10^-1^ |
| 2 | rs13004757 | 56344165 | CCDC85A | MIR216B | VRK2 | 0.302 | 0.291 | 4.10x10^-6^ | 9.39x10^-4^ | 1.14x10^-3^ | 3.14x10^-1^ |
| 5 | rs792965 | 172207869 | ERGIC1 | DUSP1 | LOC100268168 | 0.078 | 0.096 | 5.16x10^-6^ | 6.44x10^-4^ | 2.73x10^-3^ | 5.52x10^-2^ |
| 1 | rs1248482 | 79092221 | NA | IFI44 | ELTD1 | 0.261 | 0.278 | 6.67x10^-6^ | 1.30x10^-4^ | 1.94x10^-2^ | 4.29x10^-1^ |
| 2 | rs12994131 | 138135345 | THSD7B | CXCR4 | HNMT | 0.289 | 0.330 | 1.27x10^-5^ | 3.39x10^-6^ | 4.60x10^-1^ | 5.20x10^-1^ |
| 1 | rs4660527 | 41594366 | NA | SCMH1 | EDN2 | 0.454 | 0.444 | 2.09x10^-5^ | 9.17x10^-4^ | 8.25x10^-3^ | 6.26x10^-1^ |
| 18 | rs11083330 | 25231071 | NA | CDH2 | MIR302F | 0.261 | 0.257 | 2.15x10^-5^ | 4.64x10^-4^ | 1.76x10^-2^ | 6.89x10^-1^ |
| 10 | rs1556716 | 112857402 | NA | ADRA2A | GPAM | 0.231 | 0.214 | 3.06x10^-5^ | 9.80x10^-4^ | 1.13x10^-2^ | 5.54x10^-1^ |
| 12 | rs1356773 | 88079409 | NA | KITLG | DUSP6 | 0.374 | 0.365 | 4.16x10^-5^ | 3.55x10^-4^ | 4.32x10^-2^ | 7.72x10^-1^ |
| 18 | rs1786776 | 33341455 | CELF4 | KIAA1328 | LOC647946 | 0.076 | 0.069 | 4.30x10^-5^ | 7.38x10^-4^ | 2.26x10^-2^ | 1.67x10^-1^ |
| 13 | rs12584748 | 90543198 | NA | LOC144776 | MIR17HG | 0.086 | 0.076 | 4.60x10^-5^ | 5.95x10^-4^ | 3.02x10^-2^ | 9.52x10^-2^ |
| 9 | rs7034534 | 25713678 | NA | TUSC1 | C9orf82 | 0.206 | 0.177 | 4.66x10^-5^ | 2.56x10^-2^ | 8.95x10^-5^ | 9.54x10^-1^ |
| 5 | rs2460175 | 90118152 | GPR98 | LYSMD3 | ARRDC3 | 0.059 | 0.052 | 4.87x10^-5^ | 2.16x10^-3^ | 7.56x10^-3^ | 6.09x10^-1^ |
| 21 | rs2833380 | 31631306 | TIAM1 | KRTAP19-8 | SOD1 | 0.175 | 0.165 | 5.03x10^-5^ | 1.93x10^-4^ | 7.94x10^-2^ | 3.45x10^-1^ |
| 17 * | rs2668622 | 41707908 | NA | KIAA1267 | LRRC37A | 0.196 | 0.220 | 5.12x10^-5^ | 6.53x10^-4^ | 3.11x10^-2^ | NA |
| 16 | rs1030248 | 63760917 | NA | CDH11 | LOC283867 | 0.241 | 0.265 | 5.21x10^-5^ | 2.30x10^-4^ | 7.81x10^-2^ | 1.36x10^-1^ |
| 8 | rs13281882 | 19471744 | CSGALNACT1 | SH2D4A | INTS10 | 0.089 | 0.082 | 5.40x10^-5^ | 8.63x10^-4^ | 2.43x10^-2^ | 1.56x10^-1^ |
| Historical Hotspot Usage (males and females, combined) | | | | | | | | | | | |
| 5 * | rs41502455 | 23608700 | NA | PRDM9 | CDH10 | 0.147 | 0.147 | 1.31x10^-8^ | 7.73x10^-7^ | 4.30x10^-3^ | 1.75x10^-1^ |
| 2 | rs17011067 | 75399532 | NA | TACR1 | FAM176A | 0.183 | 0.210 | 1.31x10^-6^ | 1.43x10^-4^ | 2.98x10^-3^ | 2.39x10^-1^ |
| 18 | rs1864309 | 55460039 | CCBE1 | LMAN1 | PMAIP1 | 0.451 | 0.418 | 1.60x10^-6^ | 4.83x10^-4^ | 9.57x10^-4^ | 7.80x10^-1^ |
| 15 | rs16972342 | 78862853 | KIAA1199 | FAM108C1 | MIR549 | 0.062 | 0.052 | 1.04x10^-5^ | 1.09x10^-2^ | 1.02x10^-4^ | 4.78x10^-1^ |
| 9 | rs1889246 | 20170680 | NA | SLC24A2 | MLLT3 | 0.103 | 0.127 | 1.26x10^-5^ | 3.73x10^-4^ | 1.20x10^-2^ | NA |
| 22 | rs7284619 | 33847673 | NA | ISX | HMGXB4 | 0.182 | 0.176 | 1.33x10^-5^ | 4.88x10^-4^ | 9.49x10^-3^ | 6.85x10^-1^ |
| 9 | rs439005 | 32049777 | NA | MIR873 | ACO1 | 0.178 | 0.165 | 1.89x10^-5^ | 1.30x10^-2^ | 1.88x10^-4^ | 5.20x10^-1^ |
| 16 | rs12711459 | 85198336 | NA | FOXL1 | FBXO31 | 0.297 | 0.315 | 2.13x10^-5^ | 1.47x10^-2^ | 1.54x10^-4^ | 6.42x10^-1^ |
| 3 | rs6440165 | 144580138 | SLC9A9 | CHST2 | C3orf58 | 0.203 | 0.203 | 3.75x10^-5^ | 2.12x10^-4^ | 5.73x10^-2^ | 3.41x10^-2^ |
| 5 | rs277990 | 70954659 | MCCC2 | BDP1 | CARTPT | 0.345 | 0.353 | 4.33x10^-5^ | 5.51x10^-3^ | 1.82x10^-3^ | 1.75x10^-1^ |
| 3 | rs4679636 | 60078066 | FHIT | C3orf67 | PTPRG | 0.055 | 0.060 | 4.66x10^-5^ | 1.30x10^-2^ | 3.71x10^-4^ | 3.26x10^-1^ |
| 3 | rs17047384 | 102933 | NA | NA | CHL1 | 0.214 | 0.196 | 4.79x10^-5^ | 1.67x10^-3^ | 1.03x10^-2^ | 6.53x10^-1^ |
| 12 | rs1564303 | 76854938 | NAV3 | E2F7 | SYT1 | 0.392 | 0.400 | 5.06x10^-5^ | 1.11x10^-2^ | 8.14x10^-4^ | 1.09x10^-1^ |
| 12 | rs2161063 | 21900208 | ABCC9 | KCNJ8 | CMAS | 0.410 | 0.417 | 6.18x10^-5^ | 3.15x10^-4^ | 6.38x10^-2^ | 5.86x10^-1^ |
| 16 | rs11859000 | 7313050 | A2BP1 | FAM86A | A2BP1 | 0.087 | 0.083 | 6.18x10^-5^ | 8.01x10^-3^ | 1.79x10^-3^ | 6.57x10^-1^ |
| 1 | rs9729667 | 78121802 | NA | FAM73A | NEXN | 0.356 | 0.322 | 6.84x10^-5^ | 3.26x10^-4^ | 6.67x10^-2^ | 8.96x10^-2^ |
| 2 | rs1965183 | 134696436 | NA | NCKAP5 | MGAT5 | 0.356 | 0.337 | 7.40x10^-5^ | 4.25x10^-4^ | 5.63x10^-2^ | 2.56x10^-1^ |
| 22 | rs849453 | 25896619 | NA | MIAT | MN1 | 0.193 | 0.173 | 7.70x10^-5^ | 3.15x10^-3^ | 8.54x10^-3^ | 5.46x10^-1^ |
| 10 | rs4107879 | 65280959 | NA | REEP3 | ANXA2P3 | 0.197 | 0.215 | 7.75x10^-5^ | 1.43x10^-2^ | 9.67x10^-4^ | NA |
| 3 | rs6776254 | 31876852 | OSBPL10 | STT3B | ZNF860 | 0.216 | 0.220 | 8.13x10^-5^ | 1.42x10^-5^ | 4.42x10^-1^ | 2.98x10^-1^ |
| 8 | rs1595207 | 87377186 | NA | SLC7A13 | WWP1 | 0.178 | 0.176 | 8.90x10^-5^ | 1.08x10^-3^ | 3.09x10^-2^ | 4.55x10^-1^ |
| Historical Hotspot Usage (males and females, combined, PRDM9 regressed) | | | | | | | | | | | |
| 15 | rs16972342 | 78862853 | KIAA1199 | FAM108C1 | MIR549 | 0.062 | 0.052 | 4.95x10^-6^ | 1.02x10^-2^ | 3.99x10^-5^ | 8.21x10^-1^ |
| 18 | rs1864309 | 55460039 | CCBE1 | LMAN1 | PMAIP1 | 0.451 | 0.418 | 5.75x10^-6^ | 8.61x10^-4^ | 2.07x10^-3^ | 3.54x10^-1^ |
| 22 | rs7284619 | 33847673 | NA | ISX | HMGXB4 | 0.182 | 0.176 | 7.55x10^-6^ | 3.66x10^-4^ | 7.03x10^-3^ | 8.47x10^-1^ |
| 12 | rs2161063 | 21900208 | ABCC9 | KCNJ8 | CMAS | 0.410 | 0.417 | 8.60x10^-6^ | 6.97x10^-5^ | 3.65x10^-2^ | 3.19x10^-1^ |
| 2 | rs17011067 | 75399532 | NA | TACR1 | FAM176A | 0.183 | 0.210 | 9.45x10^-6^ | 3.50x10^-4^ | 9.37x10^-3^ | 5.00x10^-1^ |
| 12 | rs1564303 | 76854938 | NAV3 | E2F7 | SYT1 | 0.392 | 0.400 | 2.14x10^-5^ | 3.99x10^-3^ | 1.26x10^-3^ | 1.19x10^-1^ |
| 3 | rs6440165 | 144580138 | SLC9A9 | CHST2 | C3orf58 | 0.203 | 0.203 | 2.57x10^-5^ | 2.64x10^-4^ | 3.38x10^-2^ | 5.04x10^-2^ |
| 5 | rs277990 | 70954659 | MCCC2 | BDP1 | CARTPT | 0.345 | 0.353 | 3.03x10^-5^ | 5.61x10^-3^ | 1.11x10^-3^ | 1.68x10^-1^ |
| 3 | rs4679636 | 60078066 | FHIT | C3orf67 | PTPRG | 0.055 | 0.060 | 3.48x10^-5^ | 1.12x10^-2^ | 3.14x10^-4^ | 6.67x10^-1^ |
| 2 | rs1965183 | 134696436 | NA | NCKAP5 | MGAT5 | 0.356 | 0.337 | 4.05x10^-5^ | 7.02x10^-4^ | 2.07x10^-2^ | 4.25x10^-2^ |
| 7 | rs2355100 | 11820195 | THSD7A | PHF14 | TMEM106B | 0.355 | 0.341 | 4.13x10^-5^ | 6.71x10^-3^ | 1.54x10^-3^ | 7.79x10^-1^ |
| 9 | rs439005 | 32049777 | NA | MIR873 | ACO1 | 0.178 | 0.165 | 4.26x10^-5^ | 2.39x10^-2^ | 1.97x10^-4^ | 4.76x10^-1^ |
| 3 | rs6776254 | 31876852 | OSBPL10 | STT3B | ZNF860 | 0.216 | 0.220 | 4.63x10^-5^ | 3.43x10^-5^ | 2.10x10^-1^ | 4.38x10^-1^ |
| 8 | rs1595207 | 87377186 | NA | SLC7A13 | WWP1 | 0.178 | 0.176 | 5.14x10^-5^ | 5.63x10^-4^ | 3.26x10^-2^ | 4.53x10^-1^ |
| 10 | rs4107879 | 65280959 | NA | REEP3 | ANXA2P3 | 0.197 | 0.215 | 5.31x10^-5^ | 1.20x10^-2^ | 7.61x10^-4^ | NA |
| 14 | rs7159427 | 94567940 | NA | GSC | DICER1 | 0.263 | 0.245 | 6.17x10^-5^ | 7.68x10^-4^ | 2.96x10^-2^ | 8.39x10^-1^ |
| 9 | rs7866337 | 21669452 | NA | MIR31 | MTAP | 0.162 | 0.144 | 6.94x10^-5^ | 6.12x10^-3^ | 3.52x10^-3^ | 6.63x10^-2^ |
| 16 | rs11859000 | 7313050 | A2BP1 | FAM86A | A2BP1 | 0.087 | 0.083 | 7.01x10^-5^ | 1.05x10^-2^ | 1.41x10^-3^ | 9.45x10^-1^ |
| 16 | rs1992623 | 85203281 | NA | FOXL1 | FBXO31 | 0.296 | 0.303 | 7.08x10^-5^ | 1.59x10^-2^ | 8.28x10^-4^ | 7.44x10^-1^ |
| 5 | rs6595831 | 127863541 | FBN2 | SLC12A2 | SLC27A6 | 0.099 | 0.079 | 7.49x10^-5^ | 5.82x10^-3^ | 4.37x10^-3^ | 3.15x10^-1^ |

* Previously reported associations.

Provided are the chromosome, the rs number of the SNP with the lowest p-value in the region, the gene in which the SNP falls and the closest flanking genes. The minor allele frequencies in FHS and AGRE are given in columns «MAF (FHS)» and «MAF (AGRE)» respectively; NA applies to cases where the allele is at low frequency in the Hutterite sample or the SNP is not typed on all three arrays used for HUTT individuals. The p-values are provided for the meta-analysis of FHS and AGRE in column «P», and for FHS and AGRE separately, as well as for HUTT.

**Comparison with Chowdhury et al. (2009).** One of the phenotypes that we considered, mean recombination rate, is the same as considered by Chowdhury et al. (2009) [10] in two of the same sets of pedigrees (AGRE and FHS). Specifically, Chowdhury et al. performed a genome-wide association study of a set of AGRE families that overlap almost completely with those studied here (the overlap is 836 out of our 839 parents), then tested for replication in a very similar set of FHS individuals (1426 out of 1464 parents). They reported four new loci to be associated with total genetic map length: genes *NUB1* and *UGCG* in males and *PDZK1* and *KIAA1462* in females. Their filters were not identical to ours and their calling algorithm was implemented independently of ours. Nonetheless, we would expect excellent concordance. In two cases, our p-values from the meta-analysis are highly similar, but in two cases they are not. To identify the source of the discrepancy, we tested for an association in a series of steps: using their phenotype with our genotypes, then redoing the test but excluding any individuals that should not have been included in their study (i.e., parents of twins or individuals with a mislabeled sex), then excluding apparent phenotypic outliers in their data (i.e., 3 AGRE males and 12 FHS males with more than 40 crossovers and 5 AGRE females and 8 FHS females with more than 60 crossovers) and finally, using the first two principal components (informative of ancestry) as covariates. The results are shown in Supplementary Table 3.

**Supplementary Table 3**: A reanalysis of the top associations found by Chowdhury et al.

| Phenotypes |  |  | Ours | Chowdhury's | Chowdhury's | Chowdhury's excluding wrong sex, parents of twins, and outliers | Chowdhury's excluding wrong sex and parents of twins, and using PCs as covariates | Chowdhury's excluding wrong sex, parents of twins and outliers, and using PCs as covariates |
| --- | --- | --- | --- | --- | --- | --- | --- | --- |
| Genotypes |  |  | Ours | Chowdhury's | Ours | Ours | Ours | Ours |
|  | rs# | Gene | p-value | p-value | p-value | p-value | p-value | p-value |
| Maternal | rs1797052 | PDZK1 | **5.41 x10^-3^** | 2.34x10^-5^ | 2.89x10^-5^ | **5.02x10^-4^** | 2.77x10^-5^ | **6.38 x10^-4^** |
| Maternal | rs2505089 | KIAA1462 | 5.92x10^-6^ | 4.42x10^-7^ | 3.44x10^-7^ | 2.14x10^-6^ | 1.01x10^-7^ | 1.06x10^-6^ |
| Paternal | rs11764733 | NUB1 | 1.75x10^-6^ | 3.39x10^-6^ | 2.65x10^-5^ | 1.16x10^-5^ | 2.14x10^-5^ | 9.34x10^-6^ |
| Paternal | rs7863596 | UGCG | **1.42 x10^-2^** | 1.51x10^-7^ | 5.66x10^-7^ | **1.94x10^-3^** | 1.37x10^-7^ | **1.31 x10^-3^** |

As can be seen, when outliers are excluded, the SNPs near PDZK1 and UGCG are no longer significant in Chowdhury et al.'s analysis. Importantly, 24 of the 28 outlier individuals are not outliers in our phenotype estimates (see Supplementary Figure 9) by the Coop et al. method (or, for FHS, by the HMM estimation method). Finally, we note that although Chowdhury et al. replicate the association of RNF212 with male rate, their lowest p-value is 8.25x10^-8^ while in our analysis it is 10^-15^. Based on these comparisons, our interpretation is that two of the loci reported in Chowdhury et al. are spurious associations, driven by errors in the recombination calls.

**Supplementary Figure 9**: Mean recombination rates estimated in our study compared to

those inferred by Chowdhury et al.

Circles are AGRE parents and triangles are FHS parents. Colored points indicate parents of note: in red are parents of monozygotic twins, in which we excluded one twin from the phenotype estimation but Chowdhury et al. did not. In green are individuals whose sex was listed incorrectly, plotted with the distribution for the incorrect sex as used in Chowdhury et al. In blue are cases that we considered outliers in the Chowdhury et al. data (because their phenotypes in Chowdhury et al. are far outside the range of rates seen in previous studies, i.e., > 60 in females and >40 in males). Note that only 4 of these cases (in light blue) are also outliers by our estimation method.

**A new method to infer crossover events.** We developed a new method to infer the locations of crossover events in a nuclear family with *s* children (*s* > 1) and compared the results to those obtained using the approach of Coop et al. [9]. Let *C={C^1^,..., C^s^}* denote the observed genotypes of the *s* children and *P=(P^m^ ,P^p^)* denote the observed genotypes of the two parents (maternal and paternal). The overall strategy was to first estimate the most probable configuration of the haplotypes for the parents given the observed genotype data; then conditional on these estimated haplotypes, to reconstruct the most likely crossover event sequences independently in each child.

Genotypes for each SNP were coded by the counts (0, 1 or 2) of an arbitrarily chosen reference allele. Let *m^0^, m^1^, p^0^, p^1^* denote the (unobserved) haplotypes of mother and father (for notational convenience, *H = {m^0^, m^1^ , p^0^, p^1^}*). Let *Z^m^, Z^p^* be the vectors of indicators of a child’s maternal and paternal haplotype compositions. That is, each entry of *Z^m^ , Z^p^* takes values of {0, 1} and for example $Z_{j}^{m}=0$ means that the child’s maternal allele at the j-th locus is descended from *m^0^* . (The superscript of *Z* is dropped when we refer to arbitrary parents.) Each *Z* vector is assumed to follow a Markov process, with Pr(*Z_1_* = 0) = 0.5, and transition matrix *Qij*:=Pr(*Zn*=*j*|*Zn−1*= *i*), given by

j

$Q=\left(\begin{matrix} 1-\rho_{n} & \rho_{n} \\ \rho_{n} & 1-\rho_{n} \end{matrix}\right)$ (1)

where *ρ_n_* is the probability (per meiosis) of crossover occurring between markers *n* − 1 and *n*. Denote *ρ* = (*ρ_1_ ,. .., ρ_M_*), where *M* is the number of SNPs. In practice, we treated these values of *ρ* as known, relying on the Kong et al. (2002) genetic map [13].

We assumed that conditional on parental haplotypes *H* , children are independent, with $\Pr(C^{i}|H,\rho)=\sum_{Z^{m},Z^{p}} \Pr(C^{i}|H,Z^{m},Z^{p})\Pr(Z^{m}|\rho)\Pr(Z^{p}|\rho)$ (2)

with the standard hidden Markov assumption that the elements of C are independent given the *Z*: $\Pr(C^{i}|H,Z^{m},Z^{p})=\prod_{j} \Pr(C_{j}^{i}|H,Z_{j}^{m},Z_{j}^{p})$ (3)

where $\Pr(C_{j}^{i}|H,Z_{j}^{m},Z_{j}^{p})$ are usually referred to as the “emission probabilities”of the HMM. We assumed that these emission probabilities are given by

$\Pr(C_{j}^{i}|H,Z_{j}^{m},Z_{j}^{p})=\left\{\begin{matrix} 1-\varepsilon\text{ } \\ \varepsilon\text{ } \\ 1\text{ } \end{matrix}\begin{matrix} \text{if j-th locus is compatible} \\ \text{if j-th locus is not compatible} \\ \text{if }C_{j}\text{ is missing} \end{matrix}\right.$ (4)

where compatibility means that $m_{j}^{Z_{j}^{m}}+f_{j}^{Z_{j}^{p}}=C_{j}$ (and we assumed the jth locus is compatible if genotypes of both parents are missing at that locus). The motivation for the parameter is to account for genotyping error: we set ε = 0.001 by default, although we found that in some families with high proportions of missing genotypes at one parent and one child higher values of (e.g., equal to 0.1) produced more reasonable results (as without this higher value, the model can infer an unreasonably large number of crossovers in regions with low quality data). Although the sum (2) contains a very large number of terms, it can be computed eﬃciently using the standard “forward algorithm” for HMMs.

Given the above, the first step of our algorithm is to find the most probable parental haplotype configuration given the observed genotype data. In doing this, we considered each nuclear family separately, and so assumed that a priori all parental haplotypes are equally likely. The posterior distribution of H is then

$\Pr(H|C,P,\rho)\propto\prod_{i=1}^{s} \Pr(C^{i}|H,\rho)$ (5)

for those *H* that are compatible with the observed parental genotypes *P* (and 0 for other *H*). As noted above the probabilities $\Pr(C^{i}|H,\rho)$, given by (2), can be computed eﬃciently via the “forward algorithm”. Conditional on *H* , the *Z* have distribution

$\begin{matrix} \Pr(Z^{m},Z^{p}|H,C^{i},\rho)\propto\Pr(C^{i}|H,Z^{m},Z^{p})\Pr(Z^{m})\Pr(Z^{p}) \\ \text{ }=\prod_{j=1}^{M} \Pr(C_{j}^{i}|H,Z_{j}^{m},Z_{j}^{p})\Pr(Z^{m})\Pr(Z^{p}) \end{matrix}$ (6)

The most probable value for Z in Equation (6) is called the “Viterbi path” and its computation is standard for a hidden Markov model. We identified crossover events by computing this Viterbi path and locating the locations of switches in *Z^m^* and *Z^p^*.

Finally, we briefly describe the search algorithm that we used to attempt to find the most probable value for *H* (the posterior mode of (5)). We used an MCMC- based approach known as the STEEP algorithm from [20] to perform this optimization. In brief, STEEP combines elements of both Metropolis-coupled Markov chain Monte Carlo [21], and the Equal Energy sampler [22]. It employs multiple Markov chains that sample a sequence of target distributions in the form $\pi_{t}(x)\propto\pi(x)^{\frac{1}{t}}$. A larger *t* corresponds to a flatter (and hence easier to sample from) distribution and a smaller *t* corresponds to a steeper distribution. When *t* becomes very small, samples collected tend to be close to the global maxima. Thus, using $\Pr(H|C,P,\rho)$ as the target distribution *π*, and running a parallel series of chains with decreasing values of *t*, provides a way to perform the optimization we require. The main idea behind STEEP is that, in the MCMC sampler that updates $\pi_{t}(\cdot)$, it uses a randomly chosen sample from a higher temperature chain as its proposal. In this way, any mode that is found by a higher-temperature chain can be propogated down to lower-temperature chains, whose samples concentrate more near the global maximum.

The main requirement to use STEEP is to define the local proposal within each chain, that is a way to propose small changes to values for the parental haplotype configuration that can then be accepted or rejected. We did this as follows. First, we randomly picked a child. Next, we identified SNPs at which the phase in the child’s genotypes could be inferred unambiguosly. Specifically, for each child, its phase is unambiguous at loci that are parental recombination informative markers (PRIM) and heterozygous for the child. PRIMs are loci that are homozygous in one of the parents and heterozygous in the other. For example, suppose the father’s genotype is AA and the mother’s is AG; if a child is AG then the G allele must come from the mother (unless there are genotyping errors and/or de novo mutations). Then we dealt with each parent in turn. For each parent, we randomly chose one of its current haplotypes, and scored each PRIM at which the child is heterozygous according to whether the haplotype is the same (0) or diﬀerent (1) from the haplotype that the child inherited from that parent. This yields a vector of 0s and 1s, with one entry for each PRIM at which the child is heterozygous. We then smoothed this vector of 0s and 1s using a moving average, and identified regions containing a proportion of 1s. We selected one of these regions at random, and proposed to switch the parental haplotype at all markers that lie within that region. We then repeated this process for the second parent. Intuitively, the idea is to identify regions flanked by apparent crossover events in the randomly-selected child, and to propose to switch the parental haplotypes to remove those crossovers. Of course, this switch may induce crossovers in other children; the Metropolis-Hastings acceptance probability accounts for these in deciding whether to accept the proposed move. In addition to this local proposal, we also used longer-range proposals by compounding randomly many local proposals. In practice, we found this approach to be eﬀective in cases where genotypes are not missing, although it did not always perform well in the occasional cases where large numbers of genotypes were missing.

After calling crossover events using the Viterbi path output, we compared the results to those obtained by the method of [9]. We did so for a number of prior distributions on the recombination rate (including a fixed rate of 10^-8^ per base pair, the Kong et al. (2002)[13] recombination rates, and the Kong et al. 2002 recombination rates multiplied by a scalar) and different genotyping error rates. Shown in Supplementary Figure 10 is the correlation between methods in the mean number of crossover events per individual, for one choice of genotyping error rate and recombination rate prior. As can be seen, results from the two approaches are highly concordant, providing greater confidence in our ability to reliably infer crossovers from these data.

**Supplementary Figure 10**: Concordance between the mean number of crossovers per individual inferred by the two methods.


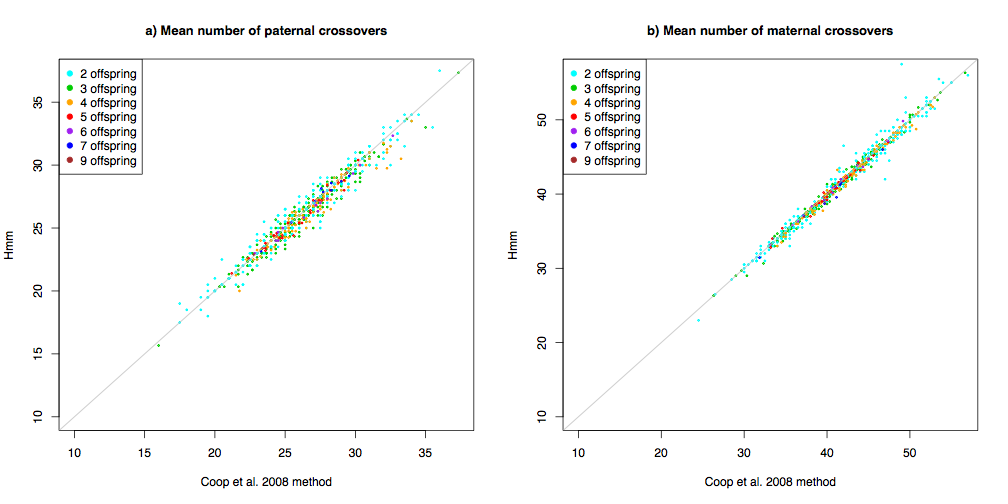


For the HMM, we used a genotyping error rate of 0.05 and a prior on the recombination rate taken from the Kong et al. genetic map multiplied by 10^-3^ per base pair.

We performed a number of additional analyses, which lent further support to this conclusion. For example, we considered the fraction of events called by either method that were called by both. To do so, we defined events as the same if they fell in overlapping intervals. We found that 98% of events that were called by the Coop et al. method were called by both methods and 98.6% of events called by the HMM were called by both. Of the events that did not overlap, 99.9 % occurred within 5cM (or 18.5 Mb) of the closest event called by the other method. Among reasons for the remaining discrepancies are: (i) Cases of families with three children, where one method assigns the crossover to one offspring whereas the other method assigns two crossovers to the same location but to the two other offspring. 2) Cases of families with four children, in which a crossover is inferred to have occurred in the same interval in two of the four children, and where each method chooses which two differently. 3) Events close to telomeres or centromeres, where there are an insufficient number of informative markers for the Coop et al. method to make a call but where the HMM method infers a crossover is likely.

**Power simulations.** We assessed our power to detect an association in the FHS, AGRE and combined sample by an approach that captures the effect of both intra-individual noise (due to variation among offspring in the same family) and inter-individual noise inherent in estimating recombination phenotypes per family. First, we resampled families (with replacement) to create a dataset with the same family size distribution. We then simulated a genotype for a parent at a SNP based on the frequency of the SNP and Hardy-Weinberg proportions, and added a fixed additive effect for this genotype onto the phenotype for the individual (the effect might be more realistically modeled as random, but it should not matter much, as we do not expect it to contribute much to the overall noise). We then tested for an additive effect of the genotype on the phenotype in a linear model framework. To assess the power to detect an association to genome-wide significance, we tabulated the fraction of simulation p-values below our cutoff of 2x10^-7^. These simulations rely on the assumption that the causal variant is among our SNPs or very well tagged by them, which should be true for approximately two-thirds of the genome (for which r^2^ > 0.8) [23,24]; if not, power will be lower.

In Supplementary Figure 11, we plot the sex-specific power to detect an allele at 20% frequency across a range of effect sizes on mean recombination rates for the FHS, AGRE and combined sample. By way of comparison, the marker in RNF212 most strongly associated with genome-wide recombination rates in males is at ~20% in the Icelandic population, and has an additive effect of ~92 cM in females and ~66 cM in males [25].

**Supplementary Figure 11**: Power to detect effects on mean recombination rate in males and females, for different effect sizes.

In males, we expect good power to detect an effect the size of RNF212 to genome-wide significance. In females, our power is much lower, as there is greater inter- and intra- individual variation. However, we have similar power to detect a marker that explains the same *proportion* of the variance in males and females (results not shown).

For comparison, Supplementary Figure 12 shows results of a similar power simulation for hotspot usage, combining both sexes, for the Framingham, AGRE and combined sample. With the combined sex sample, there is good power to detect alleles at 20% frequencies even when they have relatively subtle effects on hotspot usage.

**Supplementary Figure 12**: Power to detect effects on hotspot usage in the two sexes combined, for different effect sizes.


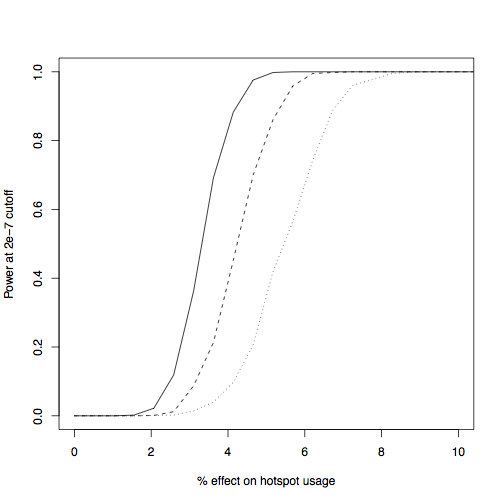


As in Supplementary Figure 11, the three lines are simulated power curves for combined, FHS and AGRE data sets (moving left to right, respectively).

**Testing for enrichments of signals**

I. We tested for an enrichment of our association signals in a list of 79 genes involved in meiotic recombination, which was compiled and kindly provided to us by Neil Hunter (University of California, Davis). For each phenotype, we found the lowest p-value that fell within 50 kb for each of these genes. We then considered the fraction of these minimum p-values that fell below some cutoff. We created a null distribution for this fraction by repeatedly sampling 79 genes at random. There was no significant enrichment (at a 0.01 or 0.05 cutoff) for our three phenotypes. In Supplementary Table 4, we provide the genes from this list that had at least one SNP with a p-value < 10^-3^.

We excluded PRDM9 and RNF212 from these lists, as these genes were identified by previous association studies and so do not represent novel findings. Including these genes in the enrichment analysis led to significant enrichments being observed at the 0.05 level.

II. We tested for an enrichment of our top signals from the meta-analysis in the HUTT data set. Specifically, we calculated the fraction of SNPs that had a p-value below a cutoff α. None of our phenotypes showed an enrichment above the expected fraction α (for a range of α  in 0.001−0.05). However, the HUTT is small and so we lack power.

III. Finally, we tested for an enrichment of SNPs associated with multiple recombination phenotypes, using the same procedure as in II above. In no comparison between phenotypes did we observe an enrichment.

**Supplementary Table 4**: Strongest associations near genes previously known to play a role in recombination from studies in model organisms

| Gene | Chromosome | Lowest P-val. | rs number | SNP position |
| --- | --- | --- | --- | --- |
| Hotspot Usage | | | | |
| PRDM9 | 5 | 1.3x10^-8^ | rs41502455 | 23608700 |
| RTEL1 | 20 | 0.0008372 | rs2872810 | 57832285 |
| Male Rate | | | | |
| SENP1 | 12 | 0.0005684 | rs11168421 | 46804600 |
| RNF212 | 4 | 4.45x10^-16^ | rs11939380 | 1076871 |
| Female Rate | | | | |
| PIAS1 | 15 | 0.0008281 | rs11071975 | 66116498 |
| RNF212 | 4 | 0.0002266 | rs1010342 | 1058819 |

The positions were determined using hg18.

**References**

1. Dawber TR, Meadors GF, Moore FE, Jr. (1951) Epidemiological approaches to heart disease: the Framingham Study. Am J Public Health Nations Health 41: 279-281.

2. Suchindran S, Rivedal D, Guyton JR, Milledge T, Gao X, et al. Genome-wide association study of Lp-PLA(2) activity and mass in the Framingham Heart Study. PLoS Genet 6: e1000928.

3. Purcell S, Neale B, Todd-Brown K, Thomas L, Ferreira MA, et al. (2007) PLINK: a tool set for whole-genome association and population-based linkage analyses. Am J Hum Genet 81: 559-575.

4. Geschwind DH, Sowinski J, Lord C, Iversen P, Shestack J, et al. (2001) The autism genetic resource exchange: a resource for the study of autism and related neuropsychiatric conditions. Am J Hum Genet 69: 463-466.

5. Patterson N, Price AL, Reich D (2006) Population Structure and Eigenanalysis. PLoS Genet 2: e190.

6. Pichler I, Fuchsberger C, Platzer C, Caliskan M, Marroni F, et al. (2010) Drawing the history of the Hutterite population on a genetic landscape: inference from Y-chromosome and mtDNA genotypes. Eur J Hum Genet 18: 463-470.

7. Ober C, Weitkamp LR, Cox N, Dytch H, Kostyu D, et al. (1997) HLA and mate choice in humans. Am J Hum Genet 61: 497-504.

8. Baudat F, Buard J, Coop G, Grey C, Ober C, et al. (2010) PRDM9 zinc finger tandem array determines meiotic recombination hotspots in humans and mice. Science In press.

9. Coop G, Wen W, Ober C, Pritchard J, Przeworski M (2008) High resolution mapping of crossovers reveals extensive variation in fine-scale recombination patterns among humans. Science 319: 1395-1398.

10. Chowdhury R, Bois PR, Feingold E, Sherman SL, Cheung VG (2009) Genetic analysis of variation in human meiotic recombination. PLoS Genet 5: e1000648.

11. Khil PP, Camerini-Otero RD Genetic crossovers are predicted accurately by the computed human recombination map. PLoS Genet 6: e1000831.

12. Baudat F, Buard J, Grey C, Fledel-Alon A, Ober C, et al. (2010) PRDM9 is a major determinant of meiotic recombination hotspots in humans and mice. Science 327: 836-840.

13. Kong A, Gudbjartsson DF, Sainz J, Jonsdottir GM, Gudjonsson SA, et al. (2002) A high-resolution recombination map of the human genome. Nat Genet 31: 241-247.

14. Myers S, Bottolo L, Freeman C, McVean G, Donnelly P (2005) A fine-scale map of recombination rates and hotspots across the human genome. Science 310: 321-324.

15. Myers S, Freeman C, Auton A, Donnelly P, McVean G (2008) A common sequence motif associated with recombination hot spots and genome instability in humans. Nat Genet.

16. Myers S, Bowden R, Tumian A, Bontrop RE, Freeman C, et al. (2010) Drive against hotspot motifs in primates implicates the PRDM9 gene in meiotic recombination. Science 327: 876-879.

17. Abney M, McPeek MS, Ober C (2000) Estimation of variance components of quantitative traits in inbred populations. Am J Hum Genet 66: 629-650.

18. Broman KW, Murray JC, Sheffield VC, White RL, Weber JL (1998) Comprehensive human genetic maps: individual and sex-specific variation in recombination. Am J Hum Genet 63: 861-869.

19. Abney M, Ober C, McPeek MS (2002) Quantitative-trait homozygosity and association mapping and empirical genomewide significance in large, complex pedigrees: fasting serum-insulin level in the Hutterites. Am J Hum Genet 70: 920-934.

20. Guan Y, Stephens M (2010) Small world MCMC with tempering: ergodicity, spectral gap and applications.: Submitted.

21. Geyer CJ, Thompson EA (1995) Annealing Markoc chain Monte Carlo with applications to ancestral inference. Journal of the American Statistical Association 90: 909-920.

22. Kou SC, Zhou Q, Wong WH (2006) Equi-energy sampler with applications in statistical inference and statistical mechanics, with discussion. Annals of Statistics 34: 1581-1619.

23. Barrett JC, Cardon LR (2006) Evaluating coverage of genome-wide association studies. Nat Genet 38: 659-662.

24. Pe'er I, de Bakker PI, Maller J, Yelensky R, Altshuler D, et al. (2006) Evaluating and improving power in whole-genome association studies using fixed marker sets. Nat Genet 38: 663-667.

25. Kong A, Thorleifsson G, Stefansson H, Masson G, Helgason A, et al. (2008) Sequence variants in the RNF212 gene associate with genome-wide recombination rate. Science 319: 1398-1401.
